# Bisphenol A and its alternatives bisphenol S and F exposure with serum uric acid levels, hyperuricemia, and gout prevalence among US adults: a nationally representative cross-sectional study

**DOI:** 10.1186/s12889-024-17883-6

**Published:** 2024-02-05

**Authors:** Shunli Jiang, Yongxin Wang, Zengliang Wang, Yaru Xu, Xi Li, Mingjia Sun, Bo Li

**Affiliations:** 1 Department of Public Health, Jining Medical University, Jining, Shandong China; 2 Department of Neurosurgery Center, The First Affiliated Hospital of Xinjiang Medical University, Urumqi, Xinjiang China; 3 Jining Center for Disease Control and Prevention, Shandong, China; 4 Department of Neurosurgery, Affiliated Hospital of Jining Medical University, Jining, China

**Keywords:** Bisphenol, Uric acid, Hyperuricemia, Gout, NHANES

## Abstract

**Background:**

Recent studies suggested inconclusive associations between bisphenols exposure and hyperuricemia risk. Our objective was to assess the potential association of bisphenol A (BPA) and its substitutes bisphenol S and F (BPS and BPF) exposure with serum uric acid (SUA) levels, hyperuricemia, and gout prevalence among US adults within the NHANES 2013-2016 datasets.

**Methods:**

Multivariable linear and logistic regression models were used to explore the associations of urinary bisphenols concentrations with SUA levels, hyperuricemia, and gout prevalence, in total population and different sex groups. The restricted cubic spline (RCS) model was used to explore the dose-response relationship.

**Results:**

In total population, doubling of urinary BPS and ∑BPs concentrations showed associations with an increase of 2.64 μmol/L (95% CI: 0.54, 4.74) and 3.29 μmol/L (95% CI: 0.59, 5.99) in SUA levels, respectively. The RCS model indicated a significantly “J”-shaped dose-response relationship between BPS exposure and SUA levels. Compared to the reference group of urinary BPS, males in the highest quartile displayed a 13.06 μmol/L (95% CI: 0.75, 25.37) rise in SUA levels. For females, doubling of urinary BPS concentrations was associated with a 3.30 μmol/L (95% CI: 0.53, 6.07) increase in SUA levels, with a significant linear dose-response relationship. In total population, doubling of urinary BPA concentrations showed a 1.05-fold (95% CI: 0.97, 1.14) adjusted risk of having hyperuricemia, with an inverted “U” curve. Doubling of urinary ∑BPs concentrations was associated with a 1.05-fold (95% CI: 0.96, 1.14) adjusted risk of hyperuricemia in total population, with a significant monotonic dose-response relationship. In females, doubling of urinary BPS concentrations was associated with a 1.45-fold (95% CI: 1.01, 2.08) adjusted increased risk of having gout, with a “J” shaped relationship.

**Conclusions:**

BPA and BPS exposure to some extent were associated with elevated SUA levels and increased risk of hyperuricemia, with different dose-response relationships and sex differences.

**Supplementary Information:**

The online version contains supplementary material available at 10.1186/s12889-024-17883-6.

## Introduction

Uric acid, derived from purine metabolism, contributed to approximately 50% of the antioxidant capacity in plasma. However, it also has harmful pro-oxidant effects including oxidative damage, inflammatory response, and endothelial dysfunction [1]. Excessive uric acid production or reduced urate excretion is an essential prerequisite for hyperuricemia and gout. Hyperuricemia and gout are risk factors for hypertension, hyperlipidemia, diabetes, kidney dysfunction, and lead to excessive premature death and disability [2, 3]. The prevalence of hyperuricemia has demonstrated an escalating trend in recent years, with rates of 11.4%, 15.1%, and 20.1% in Korea, China, and America, respectively [4–6]. Due to the increasing prevalence and severe implications, an investigation into potential risk factors of hyperuricemia and gout is imperative. Hyperuricemia is a multifaceted disease with many risk factors. Traditional risk factors such as gender, age, genetics, and lifestyles cannot entirely elucidate the magnitude and alarming spread of the hyperuricemia epidemic. Although the evidence was inconclusive, environmental pollutants including arsenic and lead had been linked to hyperuricemia and gout [7, 8].

Bisphenol A (BPA) is a highly versatile industrial compound. BPA is not only an important precursor for synthesizing polycarbonate and epoxy resin, but also can be used to produce chemical products such as plasticizers, heat stabilizers, flame retardants, coatings. BPA have been widely employed in food packaging materials, medical consumables, sports equipment, and various daily necessities [9]. Due to the wide application of BPA, it can be detected in various environmental media (water, soil, atmosphere, indoor dust, etc.) and foods [10]. In daily life, consuming food and drinking water, dermal contact with thermosensitive paper, and inhaling suspended particulate matter in the air can all cause BPA exposure [10]. However, numerous epidemiological studies have linked BPA exposure to infertility, obesity, diabetes, and cardiovascular diseases [11]. In light of the negative effects of BPA, many countries had successively imposed restrictions on its usage while proactively pursuing substitute substances. At present, bisphenol analogs are applied in chemical products, while bisphenol S (BPS) and bisphenol F (BPF) emerged as the primary alternatives to BPA. Nowadays, BPS and BPF are prevalent in various environmental media, foods, and biological samples [12]. Unfortunately, increasing literature manifested that the hormonal activities exhibited by BPS and BPF were comparable to, or even surpassed the levels of BPA [13].

Recent studies indicated that bisphenols exposure was inconclusively associated with the risk of hyperuricemia. Ma et al. revealed that BPA exposure increased uric acid synthesis via enhancing xanthine oxidase (XO) [14]. Hu et al. revealed an elevated risk of hyperuricemia development among subjects with increased serum BPA levels in China [15]. However, serum BPA had lower sensitivity than urine BPA to reflect the body burden [16]. Lee et al. conducted an assessment on the association between BPA, BPS, and BPF exposure and serum uric acid (SUA) concentrations in a sample of 489 Korean children aged 6 years. Results indicated that only BPS exposure exhibited a significant association with elevated SUA levels [14]. Hyperuricemia and gout mainly occur in adults with risk factors. However, there is limited evidence available regarding the association between bisphenols exposure and the prevalence of hyperuricemia and gout in the adult population. Herein, we conducted a nationally representative cross-sectional study to examine the relationship of BPA, BPS, and BPF exposure with SUA levels, hyperuricemia, as well as gout prevalence in the US adult population. The epidemiological evidence provided in this study will provide important clues for health risk assessment of bisphenols, as well as the prevention and treatment of hyperuricemia and gout.

## Methods

### Study population

The National Health and Nutrition Examination Survey (NHANES) is an ongoing nationally representative cross-sectional survey that uses a complex, stratified, multistage probability design to assess the health and nutritional status of the US non-institutionalized population [17]. The study protocol was approved by the research ethics review board of the National Center for Health Statistics of the Centers for Disease Control and Prevention.

Participants aged 20 years or older from NHANES 2013-2014 and 2015-2016 cycles (BPS and BPF were detected from 2013) with available information on SUA concentration (*n* = 3367), gout (*n* = 3495), urinary bisphenols and creatinine levels (*n* = 3495) were included. Considering the established links between chronic kidney disease and both hyperuricemia and gout, we excluded participants who received dialysis (*n* = 7) or had an estimated glomerular filtration rate (eGFR) less than 10 mL/min/1.73 m^2^ (*n* = 1) [7]. Finally, 3359 adults were eligible for the association of bisphenols exposure with SUA levels and hyperuricemia prevalence, while 3487 adults were eligible for the association with gout prevalence. The study protocol was approved by the research ethics review board of the National Center for Health Statistics of the Centers for Disease Control and Prevention. All participants provided written informed consent. Our study followed the guideline for strengthening the reporting of observational studies in epidemiology statement (STROBE) [18]. A STROBE checklist had been displayed in the Supplementary materials Table S1.

### Serum uric acid levels measurement, hyperuricemia and gout diagnosis

SUA concentrations were analyzed by a timed endpoint colorimetric method [19]. SUA is oxidized by the uricase to form allantoin and H_2_O_2_. The intensity of the red color formed is proportional to the concentration of SUA. The inter-assay coefficient of variation for SUA was less than 2.5% for NHANES 2013-2016. Hyperuricemia was defined as a SUA concentration exceeding 7 mg/dL in males and 6 mg/dL in females [20]. Gout has been defined as either self-reported physician diagnosis or anti-gout medication use. This is a sensitive case definition method and has been applied in extensive epidemiologic studies [7, 21]. Anti-gout medication including allopurinol, alloxanthine, colchicine, probenecid, and febuxostat use during the medical history.

### Urinary bisphenols measurement

Urine specimen was collected in urine collection cups with teflon-coated stoppers. Urinary bisphenols concentrations were analyzed by online solid phase extraction coupled to high performance liquid chromatography–isotope dilution tandem mass spectrometry with peak focusing. Further information regarding the methodology is accessible online [22]. The spiked recoveries of BPA, BPS, and BPF were 99%-104%, 104%-107%, and 91%-103%, respectively. The inter-assay coefficients of variation for the three bisphenol in quality control pools with high and low concentration levels were less than 10%. The lower limits of detection (LLOD, in μg/L) for BPA, BPS, and BPF were 0.2, 0.1, and 0.2, respectively. As NHANES protocol suggested, urine specimens with analytes concentrations below the LLOD were imputed as the LLOD divided by the square root of 2 (https://wwwn.cdc.gov/Nchs/Nhanes/2013-2014/EPHPP_H.htm). In our study, the detection rates of BPA, BPS, and BPF were 94.3%, 90.8%, and 55.6%, respectively.

In consideration of urine dilution, urinary creatinine levels were determined on a Roche Cobas 6000 Analyzer using the sarcosine oxidase method. The inter-assay coefficient of variation for urinary creatinine was less than 2.0%.

### Covariates

Information on sociodemographic (age, sex, race/ethnicity, educational attainment, income level) were collected during household interviews. Lifestyle factors (smoking, alcohol intake and physical activity), body height and weight were obtained at the mobile examination center. Race/ethnicity was categorized into non-Hispanic black, non-Hispanic white, Mexican American, and other (including multi-racial and other Hispanic group). Smoking status was categorized into never, former, and current (smoked at least 100 cigarettes during the lifetime and still smoke) [17]. Drinking status was categorized into never (had less than 12 alcohol drinks during the lifetime), former (had at least 12 alcohol drinks during the lifetime and not drink alcohol over past 12 months), as well as current mild (1 drinks/day for female, ≤ 2 drinks/day for male, or binge drinking 1 day/month), moderate (≥ 2 drinks/day for female, ≥ 3 drinks/day for male, or binge drinking ≥ 2 days/month), and heavy drinkers (≥3 drinks/day for female, ≥ 4 drinks/day for male, or binge drinking 5 days/month) [23].

Body mass index (BMI) was calculated as weight in kilograms divided by height in meters squared (kg/m^2^), and was categorized into three levels (< 25, 25-30, or ≥ 30) [17]. Physical activity was calculated according to the metabolic equivalent (MET), weekly frequency, and duration of each activity [24]. Estimated glomerular filtration rate (eGFR) was calculated according to the CKD-EPI creatinine equation [25]. Hyperlipidemia, diabetes, and hypertension were defined if participants self-reported a physician diagnosis, had deviant indexes (serum lipid, plasma glucose, or blood pressure), or current use of corresponding anti-hyperlipidemia, anti-diabetic, or anti-hypertensive medications [26–29].

### Statistical analysis

To account for the complex study design, specific subsample weights for urinary environmental phenols, stratum, and PSU variables were incorporated into all analyses. Statistical analysis was separately conducted for males and females, considering the significant disparity in their SUA levels and gout prevalence.

The associations of urinary bisphenols concentrations with SUA levels were evaluated by the coefficient and 95% confidence interval (CI) in multivariable linear regression models. The associations of urinary bisphenols concentrations with hyperuricemia and gout prevalence were assessed by multivariable logistic regression models, and the odds ratio (OR) and 95% CI were calculated. Model 1 adjusted for urinary creatinine. Model 2 further adjusted for age, sex, BMI. Model 3 further adjusted for ethnicity, smoking and drinking status, education, income, hyperlipidemia, diabetes, hypertension, and eGFR. Urinary bisphenols concentrations were respectively examined as categorical (grouped into quartiles) and continuous (log2 transformed) variables in the models. In the categorical model, the linear trend was examined by using the median value of each quartile as a continuous variable. Because BPF (55.6%) was not detected in over three quarters, an ordinal four-category variable was constructed as: individuals with BPF concentrations lower than the limit of detection were classified as the reference, individuals with detectable levels (> LLOD) were equally divided into three groups. Besides, the mass concentration of BPA, BPS, and BPF was summed to represent the total concentrations of bisphenols (∑BPs). To explore the dose-response relationship of bisphenols exposure with SUA levels, hyperuricemia, and gout prevalence, weighted restricted cubic spline (RCS) model was constructed in the “rms” R package. A covariate was incorporated into the regression model upon fulfilling any of the three criteria: a) had been selected in previous related literature; b) caused more than 10% change in the estimated exposure-effect (OR or coefficient) [30]; c) had biological relevance with hyperuricemia or gout. Urinary creatinine levels were compulsively incorporated into all models to correct urine dilution as suggested [31]. Participants with missing data on covariates were excluded from the corresponding model.

Two sensitivity analyses were designed to test the robustness of our results. First, anti-gout medication use decreases SUA concentration which might blunt bisphenols’ effects. We repeated the regression model after excluding individuals with anti-gout treatment. Second, high-purine foods and drinks are risk factors for hyperuricemia and gout. We further adjusted drinks, internal organ and seafood intake in our analyses (over 20% of participants missing data about purine intake). Bisphenols exposure was suggested to be associated with impaired renal function (decreased eGFR), which affects the excretion of uric acid and leads to hyperuricemia [32]. Hence, we assessed the mediating role of eGFR on the association between urinary bisphenol and SUA levels.

All these statistical analyses were performed on the R software (version 4.2.1). A two-tailed *P*-value < 0.05 was regarded as statistically significant.

## Results

### Characteristics of the study population

Table 1 presents the characteristics and urinary bisphenols distributions of the study population. Among these participants, 4.4% (155/3487) were diagnosed with gout, 20.6% (692/3359) were diagnosed with hyperuricemia. The mean age for the non-gout, gout, and hyperuricemia group were 46.9, 62.4, and 51.6, respectively. The median concentration of SUA in the non-gout, gout, and hyperuricemia group was 3.2, 3.9, and 4.3 μmol/L, respectively. The median concentration of BPA, BPS, and ∑BPs in gout group were higher than that in non-gout group, although statistical significance was not observed (*P* > 0.05). The concentration of BPA, BPF, and ∑BPs in hyperuricemia group were significantly higher than that in non-hyperuricemia group (*P* < 0.05).

**Table 1:** Characteristics of the study population, NHANES 2013-2016

Characteristics	Non-gout (*N* = 3332)	Gout (*N* = 155)	*P* value	Non-hyperuricemia (*N* = 2667)	Hyperuricemia (*N* = 692)	*P* value
Age, mean (SE), year	46.9 (0.5)	62.4 (1.3)	**< 0.001**	46.6 (0.5)	51.6 (0.9)	**< 0.001**
Sex			**< 0.001**			**0.005**
Female	1792 (52.5)	49 (28.7)		1465 (53.5)	320 (43.6)	
Male	1540 (47.5)	106 (71.3)		1202 (46.5)	372 (56.4)	
Race/ethnicity			0.083			**0.014**
Non-Hispanic black	735 (11.3)	39 (11.4)		551 (10.6)	177 (12.7)	
Non-Hispanic white	1202 (63.9)	73 (74.1)		970 (64.1)	270 (67.1)	
Mexican American	508 (9.1)	13 (3.8)		427 (9.5)	83 (6.9)	
Other	887 (15.7)	30 (10.7)		719 (15.8)	162 (13.4)	
Educational level			0.363			0.243
Less than high school	308 (5.2)	13 (3.1)		247 (5.0)	60 (5.4)	
High school or GED	1187 (31.3)	69 (35.8)		938 (30.3)	265 (34.6)	
College or above	1833 (63.5)	73 (61.1)		1479 (64.7)	367 (60.0)	
BMI, kg/m^2^			**0.015**			**< 0.001**
<25	1046 (33.6)	28 (17.7)		911 (36.8)	113 (16.7)	
25-30	1075 (33.1)	48 (31.2)		883 (33.8)	212 (31.3)	
≥30	1099 (33.3)	78 (51.0)		782 (29.4)	347 (52.0)	
IPR (family income to poverty ratio)			0.848			0.173
<1.30	1013 (22.7)	44 (22.1)		806 (22.7)	202 (20.4)	
1.30-3.49	1089 (34.9)	56 (32.6)		855 (33.5)	241 (38.8)	
≥3.50	921 (42.5)	43 (45.3)		763 (43.8)	181 (40.8)	
Smoking status			**0.010**			**0.020**
Never	1946 (57.3)	69 (43.4)		1577 (58.4)	375 (50.9)	
Former	714 (22.7)	67 (42.2)		555 (21.9)	191 (30.0)	
Current	667 (20.0)	19 (14.4)		530 (19.7)	126 (19.2)	
Drinking status			**0.038**			0.147
Never	491 (11.6)	19 (9.4)		390 (11.4)	99 (11.5)	
Former	509 (13.9)	37 (24.0)		396 (13.9)	129 (15.8)	
Mild	1023 (36.2)	56 (43.5)		851 (38.3)	194 (30.1)	
Moderate	444 (18.2)	12 (7.7)		353 (17.4)	94 (19.5)	
Heavy	567 (20.1)	25 (15.3)		450 (19.0)	122 (23.1)	
Physical Activity, MET-minutes/week			0.338			0.152
<600	470 (18.5)	26 (23.3)		374 (18.0)	106 (21.4)	
≥600	1990 (81.5)	76 (76.7)		1606 (82.0)	386 (78.6)	
Hyperlipidemia			**< 0.001**			**< 0.001**
No	1095 (33.7)	22 (14.4)		903 (34.7)	121 (19.1)	
Yes	2237 (66.3)	133 (85.6)		1764 (65.3)	571 (80.9)	
Diabetes			**< 0.001**			**< 0.001**
No	2665 (85.8)	90 (67.2)		2167 (87.2)	490 (75.9)	
Yes	624 (14.2)	65 (32.8)		460 (12.8)	201 (24.1)	
Hypertension			**< 0.001**			**< 0.001**
No	1942 (61.8)	29 (25.1)		1628 (64.9)	280 (43.4)	
Yes	1390 (38.2)	126 (74.9)		1039 (35.1)	412 (56.6)	
eGFR, mL/min/1.73 m^2^			**< 0.001**			**< 0.001**
<60	223 (5.9)	41 (18.4)		128 (4.0)	136 (16.2)	
60-90	994 (33.1)	72 (54.6)		800 (33.2)	265 (37.0)	
≥90	1991 (61.0)	37 (27.0)		1738 (62.7)	291 (46.7)	
BPA, μg/L	1.1 (0.6, 2.3)	1.3 (0.5, 2.8)	0.948	1.1 (0.5, 2.4)	1.2 (0.7, 2.4)	**0.032**
BPS, μg/L	0.4 (0.2, 1.0)	0.5 (0.2, 1.6)	0.529	0.4 (0.2, 1.0)	0.5 (0.2, 1.2)	0.150
BPF, μg/L	0.3 (0.1, 1.0)	0.3 (0.1, 0.8)	0.875	0.3 (0.1, 0.9)	0.3 (0.1, 1.2)	**0.038**
∑BPs, μg/L	2.7 (1.3, 5.4)	3.2 (1.3, 7.1)	0.431	2.6 (1.3, 5.3)	3.0 (1.5, 6.7)	**0.008**
Serum uric acid, μmol/L	3.2 (2.6, 3.7)	3.9 (3.2, 4.5)	**< 0.001**	3.0 (2.5, 3.4)	4.3 (4.0, 4.6)	**< 0.001**

*Abbreviations*: BMI, body mass index; eGFR, estimated glomerular filtration rate; BPA, bisphenol A; BPS, bisphenol S; BPF, bisphenol F; ∑BPs, the mass sum of the three bisphenols. Continuous variables were presented as mean (standard error) or median (25th, 75th), according to its distribution; categorical variables were presented as numbers (percentage). *P*-values were calculated by weighted Student’s t-test, Mann-Whitney *U* test, or chi-square test for different variables

### Bisphenols exposure and serum uric acid levels

**Fig. 1:**
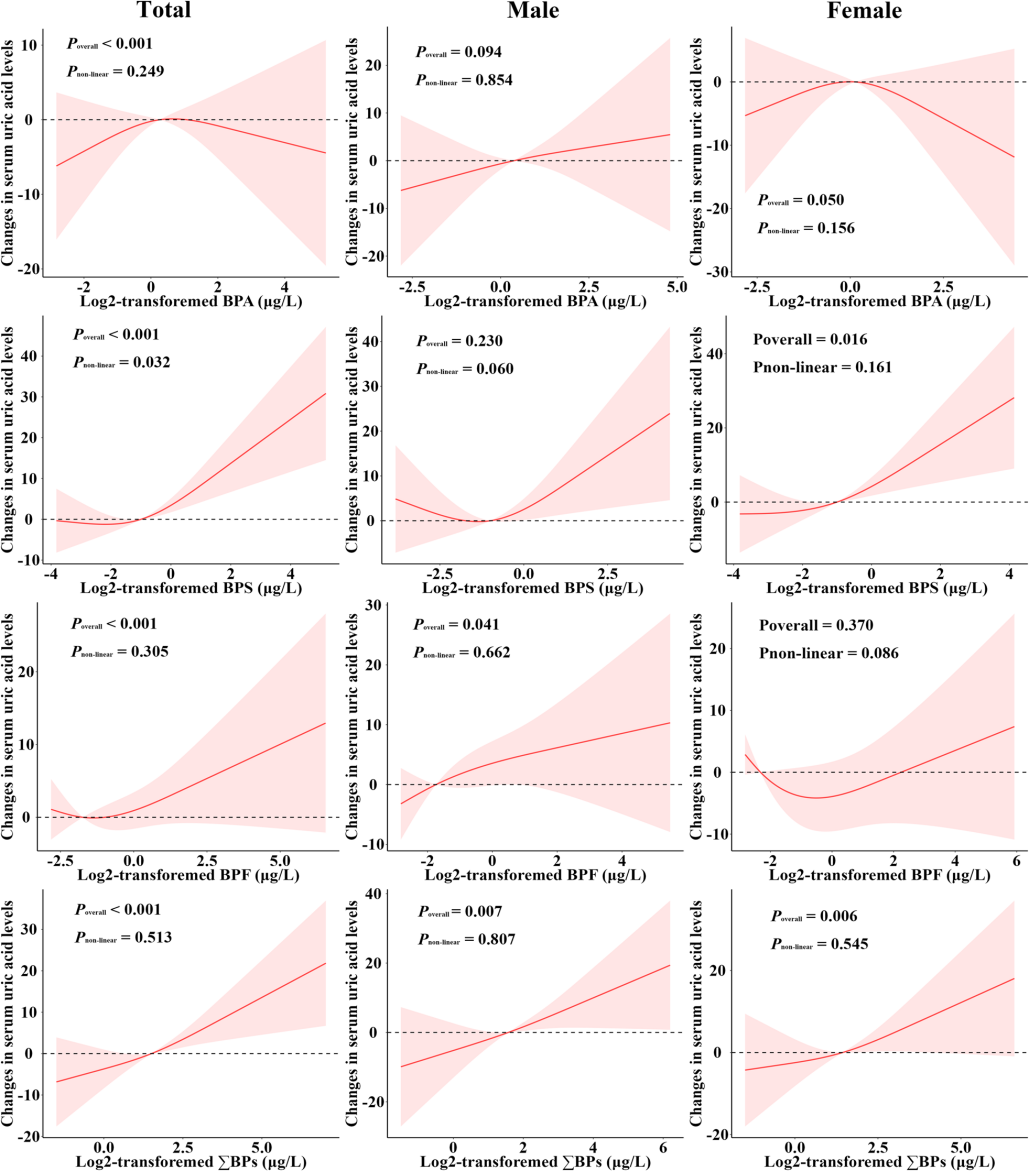
The dose-response relationships of urinary bisphenols concentrations with the changes in serum uric acid levels. Models were adjusted for urinary creatinine, age, sex, BMI, ethnicity, smoking and drinking status, education, income, hyperlipidemia, diabetes, hypertension, and eGFR

In total population, after adjusting for multiple potential confounders (Model 3), doubling of urinary BPS and ∑BPs concentrations were significantly associated with 2.64 μmol/L (95% CI: 0.54, 4.74) and 3.29 μmol/L (95% CI: 0.59, 5.99) increased uric acid levels (Table 2). In the categorical model, individuals in the highest quartile of urinary ∑BPs had a 12.02 μmol/L (95% CI: 2.71, 21.33) increased SUA levels. The RCS model indicated a significantly “J”-shaped dose-response relationship between BPS exposure and SUA levels, and a linear dose-response relationship between ∑BPs exposure and SUA levels (Fig. 1).

**Table 2:** Associations of urinary bisphenols concentrations with serum uric acid levels (μmol/L). ^a^

Outcomes	Categorical models					Continuous models	
	Quartile 1	Quartile 2	Quartile 3	Quartile 4	*P*_trend_	Doubling change	*P* value
**Total**							
BPA							
Model 1	0.00 (ref)	1.88 (–7.90, 11.66)	–1.54 (–11.27, 8.19)	–2.85 (–15.01, 9.32)	0.544	1.17 (–1.41, 3.75)	0.360
Model 2	0.00 (ref)	5.09 (–4.66, 14.83)	0.70 (–9.07, 10.48)	1.15 (–9.17, 11.47)	0.863	1.41 (–0.77, 3.60)	0.195
Model 3	0.00 (ref)	1.81 (–10.26, 13.88)	–1.13 (–13.75, 11.49)	–2.84 (–15.65, 9.97)	0.474	0.50 (–2.08, 3.08)	0.674
BPS							
Model 1	0.00 (ref)	5.13 (–6.79, 17.04)	2.72 (–12.29, 17.73)	2.74 (–12.95, 18.43)	0.115	1.85 (–0.70, 4.39)	0.148
Model 2	0.00 (ref)	3.18 (–8.65, 15.01)	–1.07 (–15.99, 13.85)	–1.89 (–16.03, 12.24)	**0.006**	**2.13 (0.26, 4.00)**	**0.027**
Model 3	0.00 (ref)	–0.08 (–14.44, 14.27)	0.02 (–15.58, 15.61)	–1.37 (–16.94, 14.21)	**0.007**	**2.64 (0.54, 4.74)**	**0.019**
BPF							
Model 1	0.00 (ref)	1.47 (–14.26, 17.19)	13.27 (–1.82, 28.37)	2.11 (–13.64, 17.87)	0.417	**1.62 (0.15, 3.09)**	**0.032**
Model 2	0.00 (ref)	0.00 (–15.52, 15.52)	7.48 (–7.31, 22.26)	–2.17 (–15.96, 11.62)	0.432	1.21 (–0.24, 2.65)	0.099
Model 3	0.00 (ref)	–3.65 (–20.45, 13.16)	3.81 (–17.00, 24.62)	–10.72 (–29.19, 7.76)	0.916	0.88 (–0.75, 2.52)	0.257
∑BPs							
Model 1	0.00 (ref)	–2.75 (–14.32, 8.82)	–0.53 (–11.41, 10.35)	6.14 (–4.38, 16.66)	0.086	**3.94 (1.65, 6.23)**	**0.001**
Model 2	0.00 (ref)	–3.64 (–13.22, 5.94)	–0.74 (–8.96, 7.47)	**8.56 (0.90, 16.22)**	0.055	**3.44 (1.33, 5.56)**	**0.003**
Model 3	0.00 (ref)	–0.21 (–10.94, 10.52)	0.15 (–8.63, 8.93)	**12.02 (2.71, 21.33)**	0.121	**3.29 (0.59, 5.99)**	**0.022**
**Male**							
BPA							
Model 1	0.00 (ref)	–3.86 (–17.11, 9.40)	4.14 (–10.38, 18.67)	5.91 (–11.21, 23.04)	0.959	2.11 (–1.36, 5.57)	0.223
Model 2	0.00 (ref)	–8.63 (–21.22, 3.96)	–0.57 (–13.51, 12.38)	4.16 (–11.59, 19.91)	0.588	1.03 (–2.16, 4.21)	0.514
Model 3	0.00 (ref)	–5.09 (–17.08, 6.91)	–0.21 (–13.72, 13.29)	8.96 (–6.60, 24.53)	0.808	1.57 (–1.90, 5.05)	0.340
BPS							
Model 1	0.00 (ref)	3.16 (–7.91, 14.23)	2.88 (–7.90, 13.66)	**15.95 (4.70, 27.19)**	0.338	1.39 (–1.96, 4.73)	0.404
Model 2	0.00 (ref)	–0.31 (–11.92, 11.29)	–2.34 (–11.59, 6.91)	**10.19 (0.68, 19.70)**	0.275	1.31 (–1.92, 4.55)	0.412
Model 3	0.00 (ref)	3.48 (–9.26, 16.22)	–0.86 (–11.77, 10.04)	**13.06 (0.75, 25.37)**	0.118	1.74 (–1.85, 5.34)	0.309
BPF							
Model 1	0.00 (ref)	8.34 (–1.46, 18.14)	5.17 (–2.81, 13.16)	6.67 (–4.08, 17.42)	0.132	**2.43 (0.29, 4.58)**	**0.027**
Model 2	0.00 (ref)	6.60 (–3.33, 16.54)	4.54 (–1.64, 10.72)	5.47 (–3.17, 14.12)	0.207	1.94 (–0.25, 4.13)	0.080
Model 3	0.00 (ref)	1.65 (–10.21, 13.51)	1.26 (–6.14, 8.66)	1.04 (–9.40, 11.48)	0.540	1.81 (–0.84, 4.47)	0.161
∑BPs							
Model 1	0.00 (ref)	6.60 (–6.87, 20.06)	9.57 (–3.19, 22.32)	12.38 (–1.00, 25.77)	0.198	**4.03 (0.93, 7.13)**	**0.013**
Model 2	0.00 (ref)	4.59 (–9.62, 18.79)	10.98 (–0.04, 22.00)	10.39 (–2.94, 23.72)	0.427	2.93 (–0.12, 5.99)	0.059
Model 3	0.00 (ref)	1.53 (–14.35, 17.41)	9.67 (–2.19, 21.54)	6.51 (–10.47, 23.49)	0.449	3.83 (–0.50, 8.15)	0.077
**Female**							
BPA							
Model 1	0.00 (ref)	9.48 (–2.78, 21.75)	3.61 (–10.57, 17.79)	3.01 (–10.54, 16.57)	0.996	2.75 (–0.22, 5.71)	0.068
Model 2	0.00 (ref)	8.49 (–4.78, 21.77)	–0.61 (–13.11, 11.88)	–1.25 (–13.47, 10.97)	0.580	1.35 (–1.32, 4.02)	0.309
Model 3	0.00 (ref)	2.90 (–11.55, 17.34)	–6.82 (–22.77, 9.12)	–6.59 (–19.75, 6.58)	0.145	–0.55 (–4.14, 3.04)	0.741
BPS							
Model 1	0.00 (ref)	5.35 (–4.44, 15.15)	4.43 (–7.30, 16.15)	11.82 (–0.74, 24.38)	**0.005**	**3.86 (1.28, 6.44)**	**0.005**
Model 2	0.00 (ref)	5.06 (–4.43, 14.54)	5.28 (–3.69, 14.26)	**12.03 (0.90, 23.16)**	**0.011**	**2.55 (0.17, 4.94)**	**0.037**
Model 3	0.00 (ref)	5.24 (–6.97, 17.45)	2.92 (–9.00, 14.84)	10.64 (–3.37, 24.65)	**0.021**	**3.30 (0.53, 6.07)**	**0.024**
BPF							
Model 1	0.00 (ref)	–5.51 (–18.13, 7.10)	7.21 (–8.68, 23.10)	6.87 (–9.35, 23.08)	0.929	1.02 (–1.42, 3.45)	0.400
Model 2	0.00 (ref)	–3.75 (–16.33, 8.83)	6.62 (–8.69, 21.94)	4.60 (–11.44, 20.63)	0.587	0.45 (–1.89, 2.79)	0.696
Model 3	0.00 (ref)	–0.57 (–14.52, 13.37)	9.41 (–7.10, 25.92)	6.67 (–12.44, 25.79)	0.251	–0.26 (–2.7, 2.19)	0.822
∑BPs							
Model 1	0.00 (ref)	8.80 (–7.05, 24.66)	9.38 (–4.60, 23.36)	**20.75 (5.24, 36.27)**	**0.017**	**5.13 (1.90, 8.37)**	**0.003**
Model 2	0.00 (ref)	3.42 (–10.84, 17.67)	2.31 (–10.19, 14.81)	14.21 (–0.68, 29.11)	0.050	**3.74 (0.58, 6.90)**	**0.022**
Model 3	0.00 (ref)	3.02 (–17.15, 23.19)	–5.25 (–20.86, 10.35)	9.51 (–8.12, 27.13)	0.177	2.67 (–1.24, 6.58)	0.161

^a^The effect of bisphenols exposure on serum uric acid levels was expressed as the coefficient and its 95% confidence interval

Model 1 was adjusted for urinary creatinine. Model 2 was adjusted for urinary creatinine, age, sex, BMI. Model 3 was adjusted for urinary creatinine, age, sex, BMI, ethnicity, smoking and drinking status, education, income, hyperlipidemia, diabetes, hypertension, and eGFR

In males, individuals in the highest quartile of urinary BPS had a 13.06 μmol/L (95% CI: 0.75, 25.37) increased SUA levels. In females, doubling of urinary BPS concentrations was associated with a 3.30 μmol/L (95% CI: 0.53, 6.07) increased SUA levels. Doubling of urinary ∑BPs concentrations was respectively associated with a 3.83 μmol/L (95% CI: –0.50, 8.15) and 2.67 μmol/L (95% CI: –1.24, 6.58) increased SUA levels in males and females. The RCS model manifested a significantly monotonic dose-response relationship between ∑BPs exposure and SUA levels in both genders (Fig. 1).

### Bisphenols exposure and hyperuricemia prevalence

**Fig. 2:**
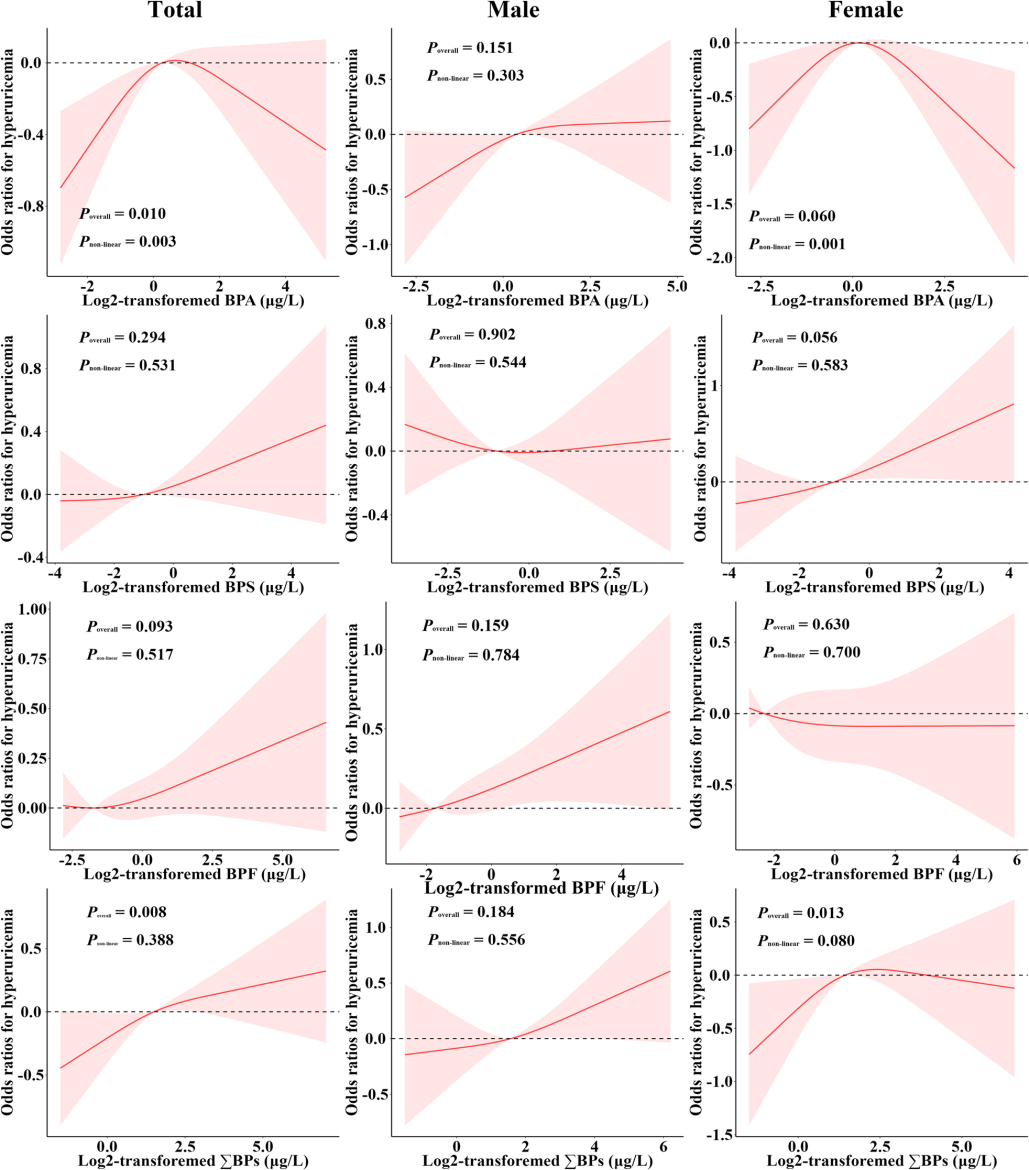
The dose-response relationships of urinary bisphenols concentrations with the risk of hyperuricemia. Models were adjusted for urinary creatinine, age, sex, BMI, ethnicity, smoking and drinking status, education, income, hyperlipidemia, diabetes, hypertension, and eGFR

In total population, doubling of urinary BPA concentrations was associated with a 1.05-fold (95%CI: 0.97, 1.14) adjusted risk of having hyperuricemia (Table 3). The RCS model manifested a significantly inverted “U” shaped non-monotonic dose-response (NMDR) relationship between BPA exposure and hyperuricemia prevalence (*P*_non-linear_ = 0.003) (Fig. 2). Doubling of urinary ∑BPs concentrations was associated with a 1.05-fold (95% CI: 0.96, 1.14) adjusted risk of having hyperuricemia in total population, with a significant monotonic dose-response relationship (Fig. 2). Doubling of urinary BPS concentrations was associated with a 1.13-fold (95% CI: 1.00, 1.28) and 1.09-fold (95% CI: 0.99, 1.21) adjusted risk of having hyperuricemia in male and female group (Table 3), with no significant dose-response relationships (Fig. 2).

**Table 3:** Associations of urinary bisphenols concentrations with hyperuricemia prevalence.^a^

Outcomes	Categorical models					Continuous models	
	Quartile 1	Quartile 2	Quartile 3	Quartile 4	*P*_trend_	Doubling change	*P* value
**Total**							
BPA							
Model 1	1.00 (ref)	**1.63 (1.17, 2.27)**	**1.46 (1.08, 1.97)**	**1.42 (1.05, 1.93)**	0.379	**1.09 (1.02, 1.16)**	**0.012**
Model 2	1.00 (ref)	**1.77 (1.27, 2.46)**	1.38 (0.98, 1.95)	**1.38 (1.04, 1.83)**	0.599	**1.07 (1.00, 1.15)**	**0.042**
Model 3	1.00 (ref)	**1.77 (1.12, 2.81)**	1.38 (0.91, 2.09)	1.31 (0.92, 1.88)	0.887	1.05 (0.97, 1.14)	0.187
BPS							
Model 1	1.00 (ref)	1.09 (0.74, 1.60)	1.08 (0.76, 1.52)	1.29 (0.90, 1.86)	0.765	**1.11 (1.01, 1.22)**	**0.035**
Model 2	1.00 (ref)	0.99 (0.64, 1.54)	0.96 (0.69, 1.33)	1.26 (0.88, 1.83)	0.783	1.08 (0.98, 1.18)	0.110
Model 3	1.00 (ref)	1.03 (0.62, 1.70)	0.91 (0.62, 1.33)	1.31 (0.81, 2.10)	0.811	1.10 (0.99, 1.23)	0.081
BPF							
Model 1	1.00 (ref)	1.17 (0.87, 1.56)	1.21 (0.88, 1.66)	1.27 (0.89, 1.82)	0.766	1.09 (1.00, 1.19)	0.052
Model 2	1.00 (ref)	1.13 (0.82, 1.57)	1.20 (0.85, 1.70)	1.24 (0.85, 1.79)	0.434	1.06 (0.96, 1.16)	0.230
Model 3	1.00 (ref)	1.03 (0.64, 1.65)	1.14 (0.71, 1.82)	1.09 (0.68, 1.74)	0.182	1.00 (0.88, 1.12)	0.926
∑BPs							
Model 1	1.00 (ref)	1.22 (0.86, 1.74)	1.34 (0.93, 1.92)	1.49 (0.97, 2.28)	0.130	1.05 (0.97, 1.14)	0.194
Model 2	1.00 (ref)	1.17 (0.79, 1.72)	1.28 (0.89, 1.84)	1.37 (0.87, 2.16)	0.090	1.05 (0.97, 1.13)	0.223
Model 3	1.00 (ref)	1.14 (0.70, 1.85)	1.18 (0.77, 1.81)	1.25 (0.72, 2.16)	0.158	1.05 (0.96, 1.14)	0.276
**Male**							
BPA							
Model 1	1.00 (ref)	**1.64 (1.02, 2.63)**	1.58 (0.94, 2.65)	1.38 (0.86, 2.21)	0.800	0.98 (0.88, 1.08)	0.652
Model 2	1.00 (ref)	**1.61 (1.00, 2.58)**	1.43 (0.84, 2.42)	1.21 (0.77, 1.92)	0.832	0.98 (0.88, 1.08)	0.632
Model 3	1.00 (ref)	1.54 (0.84, 2.83)	1.46 (0.82, 2.62)	1.30 (0.74, 2.27)	0.951	0.98 (0.88, 1.10)	0.726
BPS							
Model 1	1.00 (ref)	1.00 (0.59, 1.70)	1.03 (0.61, 1.73)	0.96 (0.56, 1.62)	**0.011**	**1.16 (1.06, 1.28)**	**0.004**
Model 2	1.00 (ref)	0.86 (0.49, 1.52)	0.90 (0.58, 1.42)	0.91 (0.54, 1.52)	**0.033**	**1.14 (1.03, 1.25)**	**0.012**
Model 3	1.00 (ref)	0.93 (0.54, 1.61)	0.89 (0.56, 1.40)	0.98 (0.58, 1.66)	0.107	1.13 (1.00, 1.28)	0.056
BPF							
Model 1	1.00 (ref)	0.92 (0.56, 1.52)	1.31 (0.76, 2.25)	1.34 (0.82, 2.18)	0.284	**1.06 (1.00, 1.12)**	**0.041**
Model 2	1.00 (ref)	0.85 (0.49, 1.48)	1.38 (0.82, 2.32)	1.29 (0.77, 2.15)	0.361	1.05 (0.99, 1.12)	0.122
Model 3	1.00 (ref)	0.86 (0.43, 1.75)	1.45 (0.77, 2.71)	1.24 (0.64, 2.38)	0.754	1.04 (0.96, 1.12)	0.294
∑BPs							
Model 1	1.00 (ref)	0.74 (0.49, 1.11)	1.13 (0.69, 1.84)	1.08 (0.62, 1.87)	0.212	**1.08 (1.00, 1.18)**	**0.048**
Model 2	1.00 (ref)	0.75 (0.47, 1.18)	1.12 (0.69, 1.83)	1.01 (0.56, 1.82)	0.280	1.07 (0.98, 1.17)	0.108
Model 3	1.00 (ref)	0.74 (0.41, 1.32)	1.15 (0.67, 1.99)	1.03 (0.51, 2.06)	0.489	1.08 (0.97, 1.21)	0.156
**Female**							
BPA							
Model 1	1.00 (ref)	1.18 (0.70, 1.98)	**1.93 (1.22, 3.05)**	1.09 (0.68, 1.74)	0.861	1.03 (0.96, 1.10)	0.377
Model 2	1.00 (ref)	1.20 (0.72, 2.02)	**1.79 (1.10, 2.91)**	0.95 (0.57, 1.60)	0.848	1.02 (0.94, 1.10)	0.631
Model 3	1.00 (ref)	1.11 (0.59, 2.07)	1.82 (0.94, 3.52)	0.76 (0.38, 1.53)	0.304	0.98 (0.89, 1.08)	0.629
BPS							
Model 1	1.00 (ref)	1.24 (0.88, 1.74)	1.27 (0.87, 1.85)	**1.94 (1.23, 3.07)**	0.104	**1.13 (1.05, 1.22)**	**0.002**
Model 2	1.00 (ref)	1.15 (0.78, 1.68)	1.10 (0.74, 1.62)	**1.73 (1.07, 2.78)**	0.221	**1.11 (1.02, 1.21)**	**0.018**
Model 3	1.00 (ref)	1.13 (0.70, 1.82)	0.93 (0.56, 1.57)	1.66 (0.87, 3.15)	0.447	1.09 (0.99, 1.21)	0.070
BPF							
Model 1	1.00 (ref)	**1.50 (1.01, 2.23)**	1.16 (0.77, 1.76)	1.16 (0.78, 1.73)	0.437	1.11 (0.99, 1.24)	0.067
Model 2	1.00 (ref)	1.55 (0.94, 2.55)	1.06 (0.67, 1.68)	1.07 (0.70, 1.63)	0.699	1.08 (0.96, 1.22)	0.188
Model 3	1.00 (ref)	1.29 (0.63, 2.61)	0.91 (0.51, 1.64)	0.80 (0.46, 1.40)	0.703	1.11 (0.96, 1.28)	0.155
∑BPs							
Model 1	1.00 (ref)	1.58 (0.91, 2.74)	1.62 (1.00, 2.63)	**2.10 (1.31, 3.37)**	**0.018**	**1.17 (1.06, 1.29)**	**0.003**
Model 2	1.00 (ref)	1.36 (0.79, 2.35)	1.28 (0.82, 2.01)	**1.83 (1.11, 3.00)**	0.059	**1.15 (1.03, 1.28)**	**0.019**
Model 3	1.00 (ref)	1.32 (0.62, 2.80)	1.05 (0.62, 1.77)	1.42 (0.75, 2.69)	0.378	1.08 (0.94, 1.24)	0.236

^a^The effect of bisphenols exposure on hyperuricemia risk was expressed as the odds ratio and its 95% confidence interval

Model 1 was adjusted for urinary creatinine. Model 2 was adjusted for urinary creatinine, age, sex, BMI. Model 3 was adjusted for urinary creatinine, age, sex, BMI, ethnicity, smoking and drinking status, education, income, hyperlipidemia, diabetes, hypertension, and eGFR

### Bisphenols exposure and gout prevalence

**Fig. 3:**
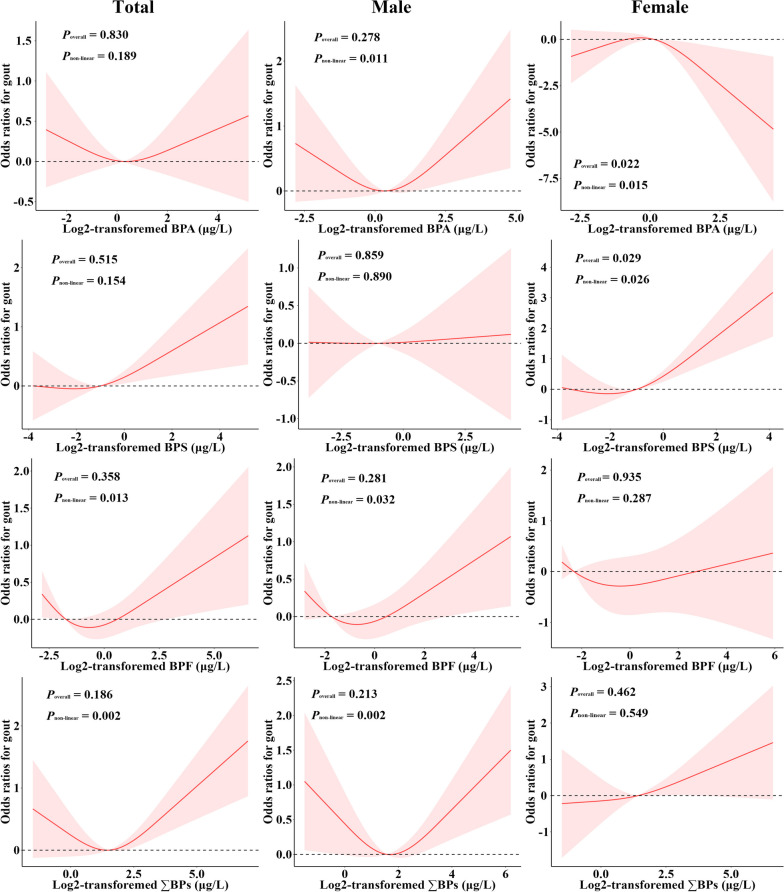
The dose-response relationships of urinary bisphenols concentrations with the risk of gout. Models were adjusted for urinary creatinine, age, sex, BMI, ethnicity, smoking and drinking status, education, income, hyperlipidemia, diabetes, hypertension, and eGFR. Color should be used for all figures in print

In total population, no significant associations and dose-response relationships between bisphenols exposure and gout prevalence were established (Table 4 and Fig. 3). In males, individuals with higher BPA levels pretended to have high gout prevalence (*P*_trend_ = 0.036), with an NMDR relationship (*P*_non-linear_ = 0.011). In females, doubling of urinary BPS concentrations was linked to a 1.45-fold (95% CI: 1.01, 2.08) adjusted increased risk of having gout, with a “J” shaped NMDR relationship (*P*_non-linear_ = 0.026).

**Table 4:** Associations of urinary bisphenols concentrations with gout prevalence. ^a^

Outcomes	Categorical models					Continuous models	
	Quartile 1	Quartile 2	Quartile 3	Quartile 4	*P*_trend_	Doubling change	*P* value
**Total**							
BPA							
Model 1	1.00 (ref)	0.71 (0.39, 1.30)	0.96 (0.43, 2.16)	1.12 (0.53, 2.40)	0.454	1.02 (0.83, 1.25)	0.886
Model 2	1.00 (ref)	0.84 (0.47, 1.52)	1.05 (0.48, 2.33)	1.26 (0.66, 2.39)	0.315	1.02 (0.86, 1.22)	0.783
Model 3	1.00 (ref)	0.78 (0.39, 1.56)	0.91 (0.35, 2.38)	1.14 (0.50, 2.57)	0.515	1.00 (0.81, 1.23)	0.987
BPS							
Model 1	1.00 (ref)	1.59 (0.78, 3.21)	0.91 (0.41, 2.01)	1.74 (0.76, 3.99)	0.312	1.09 (0.91, 1.31)	0.334
Model 2	1.00 (ref)	1.55 (0.74, 3.24)	0.92 (0.45, 1.88)	2.05 (0.88, 4.75)	0.164	1.11 (0.92, 1.33)	0.264
Model 3	1.00 (ref)	1.74 (0.75, 4.06)	0.97 (0.42, 2.26)	2.46 (0.85, 7.14)	0.137	1.14 (0.90, 1.44)	0.251
BPF							
Model 1	1.00 (ref)	0.85 (0.46, 1.55)	1.02 (0.48, 2.16)	0.86 (0.38, 1.95)	0.753	1.04 (0.89, 1.22)	0.605
Model 2	1.00 (ref)	0.78 (0.40, 1.51)	0.98 (0.45, 2.13)	0.81 (0.36, 1.82)	0.685	1.02 (0.87, 1.20)	0.768
Model 3	1.00 (ref)	0.79 (0.34, 1.83)	0.94 (0.39, 2.25)	0.79 (0.35, 1.81)	0.625	1.04 (0.88, 1.22)	0.654
∑BPs							
Model 1	1.00 (ref)	0.77 (0.45, 1.34)	0.77 (0.34, 1.72)	1.60 (0.64, 4.02)	0.117	1.15 (0.93, 1.43)	0.198
Model 2	1.00 (ref)	0.71 (0.42, 1.21)	0.74 (0.33, 1.66)	1.56 (0.62, 3.94)	0.131	1.13 (0.89, 1.42)	0.304
Model 3	1.00 (ref)	0.53 (0.23, 1.22)	0.60 (0.23, 1.54)	1.55 (0.52, 4.60)	0.101	1.16 (0.89, 1.50)	0.239
**Male**							
BPA							
Model 1	1.00 (ref)	0.51 (0.22, 1.18)	0.86 (0.27, 2.73)	1.91 (0.77, 4.76)	**0.018**	1.07 (0.82, 1.39)	0.596
Model 2	1.00 (ref)	0.60 (0.26, 1.35)	0.86 (0.27, 2.71)	2.05 (0.95, 4.44)	**0.011**	1.07 (0.85, 1.34)	0.542
Model 3	1.00 (ref)	0.52 (0.20, 1.32)	0.76 (0.21, 2.73)	2.06 (0.76, 5.62)	**0.036**	1.08 (0.82, 1.42)	0.570
BPS							
Model 1	1.00 (ref)	1.95 (0.85, 4.44)	1.02 (0.41, 2.55)	1.16 (0.51, 2.61)	0.707	0.98 (0.86, 1.13)	0.804
Model 2	1.00 (ref)	1.76 (0.71, 4.41)	0.96 (0.41, 2.26)	1.28 (0.54, 3.01)	0.992	0.99 (0.87, 1.15)	0.944
Model 3	1.00 (ref)	1.94 (0.63, 6.01)	1.14 (0.40, 3.27)	1.52 (0.49, 4.75)	0.710	1.01 (0.83, 1.23)	0.908
BPF							
Model 1	1.00 (ref)	0.92 (0.48, 1.76)	0.82 (0.35, 1.95)	0.94 (0.35, 2.48)	0.962	1.04 (0.86, 1.26)	0.687
Model 2	1.00 (ref)	0.85 (0.39, 1.82)	0.81 (0.32, 2.02)	0.86 (0.31, 2.39)	0.864	1.02 (0.84, 1.24)	0.854
Model 3	1.00 (ref)	0.84 (0.32, 2.22)	0.90 (0.28, 2.88)	0.97 (0.32, 2.92)	0.962	1.06 (0.85, 1.31)	0.596
∑BPs							
Model 1	1.00 (ref)	0.95 (0.43, 2.12)	0.55 (0.25, 1.20)	1.76 (0.63, 4.92)	0.111	1.10 (0.83, 1.46)	0.478
Model 2	1.00 (ref)	1.01 (0.46, 2.22)	0.55 (0.25, 1.23)	1.73 (0.63, 4.78)	0.156	1.07 (0.81, 1.40)	0.632
Model 3	1.00 (ref)	0.91 (0.31, 2.64)	0.52 (0.20, 1.36)	1.94 (0.52, 7.30)	0.138	1.12 (0.81, 1.55)	0.473
**Female**							
BPA							
Model 1	1.00 (ref)	1.80 (0.86, 3.78)	1.25 (0.4, 3.91)	0.65 (0.16, 2.58)	0.228	0.95 (0.78, 1.15)	0.563
Model 2	1.00 (ref)	1.79 (0.87, 3.72)	1.11 (0.37, 3.32)	0.57 (0.16, 2.07)	0.146	0.91 (0.77, 1.09)	0.300
Model 3	1.00 (ref)	1.74 (0.65, 4.63)	0.83 (0.22, 3.11)	0.44 (0.08, 2.45)	0.149	0.85 (0.66, 1.08)	0.165
BPS							
Model 1	1.00 (ref)	0.94 (0.26, 3.43)	0.95 (0.32, 2.84)	**4.50 (1.03, 19.61)**	**0.021**	**1.41 (1.01, 1.96)**	**0.043**
Model 2	1.00 (ref)	0.90 (0.25, 3.18)	0.98 (0.32, 3.02)	4.09 (0.80, 20.80)	**0.049**	1.38 (0.96, 1.97)	0.078
Model 3	1.00 (ref)	1.18 (0.25, 5.55)	0.76 (0.24, 2.42)	5.13 (0.90, 29.30)	**0.049**	**1.45 (1.01, 2.08)**	**0.043**
BPF							
Model 1	1.00 (ref)	0.65 (0.19, 2.25)	1.53 (0.56, 4.16)	0.91 (0.28, 2.94)	0.909	1.06 (0.86, 1.31)	0.598
Model 2	1.00 (ref)	0.62 (0.16, 2.32)	1.38 (0.48, 3.98)	0.85 (0.28, 2.56)	0.826	1.04 (0.85, 1.28)	0.709
Model 3	1.00 (ref)	0.59 (0.11, 3.23)	0.86 (0.29, 2.56)	0.55 (0.11, 2.70)	0.451	0.98 (0.73, 1.31)	0.861
∑BPs							
Model 1	1.00 (ref)	0.96 (0.35, 2.64)	2.32 (0.52, 10.27)	2.47 (0.43, 14.26)	0.218	1.31 (0.98, 1.74)	0.066
Model 2	1.00 (ref)	0.72 (0.25, 2.06)	1.70 (0.38, 7.53)	2.02 (0.34, 12.14)	0.271	1.26 (0.92, 1.74)	0.145
Model 3	1.00 (ref)	0.60 (0.17, 2.11)	1.29 (0.20, 8.35)	1.83 (0.32, 10.51)	0.218	1.24 (0.88, 1.75)	0.193

^a^The effect of bisphenols exposure on hyperuricemia risk was expressed as the odds ratio and its 95% confidence interval

Model 1 was adjusted for urinary creatinine. Model 2 was adjusted for urinary creatinine, age, sex, BMI. Model 3 was adjusted for urinary creatinine, age, sex, BMI, ethnicity, smoking and drinking status, education, income, hyperlipidemia, diabetes, hypertension, and eGFR

### Sensitivity and mediation analyses

The associations of bisphenols exposure with SUA levels and hyperuricemia prevalence remained robust while excluding individuals with anti-gout treatment (Table S2 and S3). After further adjusting purine intake in the regression model, BPS exposure still was positively associated with SUA levels, but the associations of ∑BPs exposure with SUA levels, hyperuricemia, and gout prevalence tended to be nonsignificant (Table S4-S6).

In total population, urinary BPS concentrations exhibited a positive association with eGFR levels (*P*_trend_ = 0.023) (Table S7). In male group, both urinary BPS and ∑BPs concentrations were positively associated with eGFR levels. However, no mediating effects of eGFR on the associations between urinary bisphenols and SUA levels were found (Table S8).

## Discussion

In the present study, urinary BPA, BPF, and ∑BPs in hyperuricemia group were significantly higher than those in non-hyperuricemia group. After adjustment for potential confounders, BPS and ∑BPs exposure exhibited a positive association with SUA levels in total and female population. BPA exposure was to some degree associated with hyperuricemia prevalence in total population with an inverted “U” shaped dose-response relationship.

In accordance with previous research, our findings indicate a positive correlation between BPA exposure and the prevalence of hyperuricemia. Ma et al. enrolled 80 patients with hyperuricemia and matched 160 subjects without hyperuricemia based on age and gender [33]. Serum BPA concentration in patients with hyperuricemia (1.83 ± 1.91 ng/mL) was significantly higher than that in non-hyperuricemia subjects (1.15 ± 1.52 ng/mL). After adjusting for confounders, serum BPA concentration was associated with a 1.93-fold (95% CI: 1.16, 2.84) increased risk of developing hyperuricemia. Furthermore, this research group designed a prospective study to confirm BPA as an independent risk factor of hyperuricemia [15]. At baseline, participants with higher serum BPA concentrations exhibited significantly elevated SUA levels. After six-year follow-up, participants within the high BPA group had a 2.42-fold (95% CI: 1.07, 5.48) increased risk of having hyperuricemia. However, those two studies both used serum BPA concentration (only reflecting the active part of BPA) to represent BPA exposure, which might underestimate the human body burden and its adverse effects [16]. Lee et al. measured urinary BPA, BPS, and BPF concentrations to reflect the body burden in children [14]. The median concentration was 1.58 μg/L for BPA, the detection rate of BPS and BPF was 41.9% and 23.5%, respectively. After adjusting for confounders, boys in the high BPS group had 0.41 mg/dL (95%CI: 0.16, 0.66) increased SUA levels. But the relatively small sample size (*n* = 489) restricted the extrapolation of the finding. In our study, significant associations were observed between BPS and ∑BPs exposure and SUA levels. Our findings along with the aforementioned studies, further substantiate the detrimental impact of bisphenols on purine metabolism.

BPS and BPF were recently introduced as alternatives to BPA, resulting in a comparable yet relatively lower body burden. In the present study, the median concentrations of BPA, BPS, and BPF in total population were 1.2 μg/L, 0.5 μg/L, and 0.3 μg/L, respectively. The median concentrations of BPA, BPS, and BPF for general individuals in Wuhan, China were 0.60 μg/L, 0.27 μg/L, and 0.26 μg/L, respectively [9]. Similar distribution also can be found in India, Japan, and other countries [34, 35]. With the volume production and widespread application of BPS and BPF as substitutes for BPA, their increased body burden and potential adverse effects deserve more attention. Although some reviews had summarized the similarities among BPA, BPS, and BPF, their disparities worth deeply exploring. The oral systemic bioavailability of BPS was 250 times greater than that of BPA [36]. BPS exerted the greatest efficacy on 17α-OH progesterone while BPF exhibited the highest efficacy on progesterone. BPS was predicted to serve as a substrate for CYP2C9, while BPA and BPF were anticipated to act as substrates for CYP3A4 [37]. Liu et al. indicated that BPF rather than BPA and BPS had the highest risk quotient on the ecosystem [38]. Thoene et al. summarized that dietary BPS exposure caused more pathologies in the reproductive system compared to BPA and other analogs [38]. Considering the equal or higher toxicity of BPS and BPF, they should be under same legal supervision as BPA.

We found an NMDR relationship between BPA exposure and hyperuricemia prevalence. Calabrese and Baldwin inferred that about 40% of the dose-response relationships within the realm of endocrine disrupters were non-monotonic [39]. The established mechanisms of NMDR include but not limited to cytotoxicity, cell and tissue specific receptors and cofactors, receptor selectivity, receptor competition, receptor down-regulation, desensitization, negative feedback loops, and other downstream mechanisms [40]. The prevailing regulations and acceptable daily intake of BPA around the world are based on an assumption of monotonicity. However, the effects observed at high doses under NMDR curves do not accurately predict the effects of low doses, which are typically encountered by the general population. There is an urgent need for fundamental reform in chemical toxicity assessment and exposure standards.

Although the precise mechanism underlying the relationship between bisphenols exposure and hyperuricemia remains uncertain, enhanced activity of XO is presumed to play a vital role. XO, a rate-limiting enzyme in the liver for uric acid synthesis, exerted increased activity in response to BPA exposure, leading to heightened uric acid levels in serum and liver, but not in urine [33]. Furthermore, the circular dichroism and molecular docking analyses indicated that BPA altered the secondary structures of XO by binding to its Asp360 and Lys422 sites. Additionally, BPA has the potential to metabolize into bisphenol A 3,4-quinone, which could potentially enhance XO activity and subsequently increase uric acid synthesis [16]. ATP-binding cassette subfamily G member 2 (ABCG2) is identified as a high-capacity urate transporter and its dysfunction is linked to elevated SUA levels and an increased risk of hyperuricemia. Some studies had found down-regulated ABCG2 protein expression and damaged function after BPA treatment [41, 42]. Decreased renal function impedes uric acid excretion, resulting in its accumulation and consequent hyperuricemia. Environmental bisphenols exposure impairs renal function by reducing kidney tubule formation, inducing ferroptosis, increasing tubular injury, and might contribute to urate underexcretion [32, 43, 44]. Besides, bisphenols-induced oxidative stress could worsen renal function and further restrict urate excretion [44, 45]. No mediating effects of eGFR were found on the associations between bisphenols exposure and SUA levels, suggesting the presence of alternative, more specific mechanisms underlying these relationships.

Sex differences were noticed in our findings. Although urinary BPS concentrations were higher in males, the relationship between BPS exposure and SUA levels, as well as the prevalence of hyperuricemia, exhibited greater significance in females. In our study, females were older and have less physical activity in comparison to males. These detrimental factors could potentially amplify the impacts of BPS on females (Table S9). Besides, the higher prevalence of hyperuricemia among males can be attributed mainly to inherent genetic variants rather than external factors [46, 47]. Additional hypothesis-driven research is needed to further understand the sex-specific effects of BPS.

The strengths of our study included standardized laboratory analysis and the nationally representative of the subjects. However, the cross-sectional study design limits the ability to determine causality in the observed associations. Nonetheless, considering the numerous documented experiments and epidemiologic studies, it is more reasonable to hypothesize that bisphenols exposure influences uric acid levels, rather than the other way around. Second, bisphenols exposure conditions were assessed only in one urine sample, potential non-differential exposure misclassification could weaken the risk estimates. Consequently, in prospective studies, utilizing repeated urine samples to ascertain bisphenols exposure levels is recommended to validate our findings.

## Conclusions

Environmental BPS and ∑BPs exposure showed positive associations with SUA levels in total and female population among US adults. The dose-response relationship between BPA exposure and hyperuricemia prevalence in total population conformed to an inverted “U” curve. Given the widespread use and adverse effects, our findings underline the need for the government to reassess the presence of bisphenols in daily life. Additional prospective studies and mechanical research are required to verify our findings and shed light on the precise mechanisms involved.

### Supplementary Information

**Additional file 1:** **Table S1.** STROBE Checklist for Cross-sectional Studies. **Table S2.** Associations of urinary bisphenols concentrations with serum uric acid levels (μmol/L), excluding individuals with anti-gout treatment. **Table S3.** Associations of urinary bisphenols concentrations with hyperuricemia prevalence, excluding individuals with anti-gout treatment. **Table S4**. Associations of urinary bisphenols concentrations with serum uric acid levels (μmol/L), adjusting drinks, organ and seafood intake. **Table S5.** Associations of urinary bisphenols concentrations with hyperuricemia prevalence, adjusting drinks, organ and seafood intake. **Table S6.** Associations of urinary bisphenols concentrations with gout prevalence, adjusting drinks, organ and seafood intake. **Table S7.** Associations of urinary bisphenols concentrations with eGFR levels. **Table S8.** Mediating effects of eGFR on the associations between urinary bisphenols and serum uric acid levels (μmol/L). **Table S9.** Sex difference of the study population, NHANES 2013-2016.

## Data Availability

The datasets presented in this study are accessible via online repositories (https://www.cdc.gov/nchs/nhanes/).
