# Supplementary material for: Bisphenol A and its alternatives bisphenol S and F exposure with serum uric acid levels, hyperuricemia, and gout prevalence among US adults: a nationally representative cross-sectional study
Source: BMC Public Health. 2024 Feb 5;24:370. doi: 10.1186/s12889-024-17883-6 (PMC10840165; doi:10.1186/s12889-024-17883-6)
Supplement: Supplementary file 1 — Additional file 1: Table S1. STROBE Checklist for Cross-sectional Studies. Table S2. Associations of urinary bisphenols concentrations with serum uric acid levels (μmol/L), excluding individuals with anti-gout treatment. Table S3. Associations of urinary bisphenols concentrations with hyperuricemia prevalence, excluding individuals with anti-gout treatment. Table S4. Associations of urinary bisphenols concentrations with serum uric acid levels (μmol/L), adjusting drinks, organ and seafood intake. Table S5. Associations of urinary bisphenols concentrations with hyperuricemia prevalence, adjusting drinks, organ and seafood intake. Table S6. Associations of urinary bisphenols concentrations with gout prevalence, adjusting drinks, organ and seafood intake. Table S7. Associations of urinary bisphenols concentrations with eGFR levels. Table S8. Mediating effects of eGFR on the associations between urinary bisphenols and serum uric acid levels (μmol/L). Table S9. Sex difference of the study population, NHANES 2013-2016. [file 12889_2024_17883_MOESM1_ESM.docx]

**Bisphenol A and its alternatives bisphenol S and F exposure with serum uric acid levels, hyperuricemia, and gout prevalence among US adults: A nationally representative cross-sectional study**

**Table of contents**

Table S1. STROBE Checklist for Cross-sectional Studies

Table S2. Associations of urinary bisphenols concentrations with serum uric acid levels (μmol/L), excluding individuals with anti-gout treatment.

Table S3. Associations of urinary bisphenols concentrations with hyperuricemia prevalence, excluding individuals with anti-gout treatment.

Table S4. Associations of urinary bisphenols concentrations with serum uric acid levels (μmol/L), adjusting drinks, organ and seafood intake.

Table S5. Associations of urinary bisphenols concentrations with hyperuricemia prevalence, adjusting drinks, organ and seafood intake.

Table S6. Associations of urinary bisphenols concentrations with gout prevalence, adjusting drinks, organ and seafood intake.

Table S7. Associations of urinary bisphenols concentrations with eGFR levels.

Table S8. Mediating effects of eGFR on the associations between urinary bisphenols and serum uric acid levels (μmol/L).

Table S9. Sex difference of the study population, NHANES 2013-2016.

Table S1. STROBE Checklist for Cross-sectional Studies

| **Section** | **Item No** | **Recommendation** | **Check (√)** | **Page and Line** |
| --- | --- | --- | --- | --- |
| **Title and abstract** | 1 | (*a*) Indicate the study’s design with a commonly used term in the title or the abstract | √ | Page 1, Line1-2 |
|  |  | (*b*) Provide in the abstract an informative and balanced summary of what was done and what was found | √ | Page 2, Line 19-36 |
| **Introduction** | | |  |  |
| Background/rationale | 2 | Explain the scientific background and rationale for the investigation being reported | √ | Page 4, Line 72-82 |
| Objectives | 3 | State specific objectives, including any prespecified hypotheses | √ | Page 4, Line 82-85 |
| **Methods** | | |  |  |
| Study design | 4 | Present key elements of study design early in the paper | √ | Page 5, Line 90-95 |
| Setting | 5 | Describe the setting, locations, and relevant dates, including periods of recruitment, exposure, follow-up, and data collection | √ | Page 5, Line 95-100 |
| Participants | 6 | (*a*) Give the eligibility criteria, and the sources and methods of selection of participants | √ | Page 5, Line 95-100 |
| Variables | 7 | Clearly define all outcomes, exposures, predictors, potential confounders, and effect modifiers. Give diagnostic criteria, if applicable | √ | Page 5-6, Line 112-117  Page 6, Line 125-129  Page 7, Line 138-154 |
| Data sources/ measurement | 8* | For each variable of interest, give sources of data and details of methods of assessment (measurement). Describe comparability of assessment methods if there is more than one group | √ | Page 6, Line 119-130 |
| Bias | 9 | Describe any efforts to address potential sources of bias | √ | Page 8, Line 176-180 |
| Study size | 10 | Explain how the study size was arrived at | √ | Page 5, Line 100-102 |
| Quantitative variables | 11 | Explain how quantitative variables were handled in the analyses. If applicable, describe which groupings were chosen and why | √ | Page 7, Line 138-146 |
| Statistical methods | 12 | (*a*) Describe all statistical methods, including those used to control for confounding | √ | Page 7-8, Line 160-181 |
|  |  | (*b*) Describe any methods used to examine subgroups and interactions | √ | Page 7, Line 158-159 |
|  |  | (*c*) Explain how missing data were addressed | √ | Page 5, Line 95-97  Page 8, Line 181 |
|  |  | (*d*) If applicable, describe analytical methods taking account of sampling strategy | √ | Page 7, Line 156-159 |
|  |  | (*e*) Describe any sensitivity analyses | √ | Page 8, Line 182-187 |
| **Results** | | |  |  |
| Participants | 13* | (a) Report numbers of individuals at each stage of study—eg numbers potentially eligible, examined for eligibility, confirmed eligible, included in the study, completing follow-up, and analysed | √ | Page 9, Line 195-197 |
|  |  | (b) Give reasons for non-participation at each stage | √ | Page 5, Line 95-100 |
|  |  | (c) Consider use of a flow diagram |  |  |
| Descriptive data | 14* | (a) Give characteristics of study participants (eg demographic, clinical, social) and information on exposures and potential confounders | √ | Page 9, Line 195-203 |
|  |  | (b) Indicate number of participants with missing data for each variable of interest | √ | Page 5, Line 95-100 |
| Outcome data | 15* | Report numbers of outcome events or summary measures | √ | Page 9, Line 195-197 |
| Main results | 16 | (*a*) Give unadjusted estimates and, if applicable, confounder-adjusted estimates and their precision (eg, 95% confidence interval). Make clear which confounders were adjusted for and why they were included | √ | Page 9, Line 205-214  Page 14-19 |
|  |  | (*b*) Report category boundaries when continuous variables were categorized | √ | Page 9, Line 208-209 |
|  |  | (*c*) If relevant, consider translating estimates of relative risk into absolute risk for a meaningful time period |  |  |
| Other analyses | 17 | Report other analyses done—eg analyses of subgroups and interactions, and sensitivity analyses | √ | Page 11, Line 238-242 |
| **Discussion** | | |  |  |
| Key results | 18 | Summarise key results with reference to study objectives | √ | Page 23, Line 260-264 |
| Limitations | 19 | Discuss limitations of the study, taking into account sources of potential bias or imprecision. Discuss both direction and magnitude of any potential bias | √ | Page 26, Line 341-346 |
| Interpretation | 20 | Give a cautious overall interpretation of results considering objectives, limitations, multiplicity of analyses, results from similar studies, and other relevant evidence | √ | Page 26, Line 350-352 |
| Generalisability | 21 | Discuss the generalisability (external validity) of the study results | √ | Page 26, Line 353-356 |
| **Other information** | | |  |  |
| Funding | 22 | Give the source of funding and the role of the funders for the present study and, if applicable, for the original study on which the present article is based | √ | Page 27, Line 366-371 |

*Give information separately for exposed and unexposed groups.

| Table S2. Associations of urinary bisphenols concentrations with serum uric acid levels (μmol/L), excluding individuals with anti-gout treatment.^a^ | | | | | | | | | | | | | | | | | | | | | |
| --- | --- | --- | --- | --- | --- | --- | --- | --- | --- | --- | --- | --- | --- | --- | --- | --- | --- | --- | --- | --- | --- |
| Outcomes | | Categorical models | | | | | | | | | | | | | |  | | Continuous models | | | |
|  |  | Quartile 1 | | Quartile 2 | | Quartile 3 | | | | Quartile 4 | | | *P*_trend_ | | |  | | Doubling change | | *P* value | |
| **Total** | |  | |  | |  | | | |  | | |  | | |  | |  | |  | |
| BPA | |  | |  | |  | | | |  | | |  | | |  | |  | |  | |
| Model 1 | | 0.00 (ref) | | 1.59 (–8.00, 11.18) | | –1.09 (–10.84, 8.66) | | | | –3.16 (–15.61, 9.29) | | | 0.521 | | |  | | 1.04 (–1.62, 3.70) | | 0.431 | |
| Model 2 | | 0.00 (ref) | | 3.99 (–5.40, 13.380) | | 0.89 (–8.64, 10.41) | | | | 0.78 (–9.81, 11.37) | | | 0.867 | | |  | | 1.25 (–1.02, 3.52) | | 0.266 | |
| Model 3 | | 0.00 (ref) | | 0.73 (–11.17, 12.63) | | –1.35 (–14.23, 11.53) | | | | –3.32 (–16.14, 9.50) | | | 0.472 | | |  | | 0.38 (–2.22, 2.97) | | 0.751 | |
| BPS | |  | |  | |  | | | |  | | |  | | |  | |  | |  | |
| Model 1 | | 0.00 (ref) | | –2.51 (–14.41, 9.38) | | –0.61 (–11.57, 10.35) | | | | 5.69 (–5.14, 16.52) | | | 0.156 | | |  | | 1.78 (–0.86, 4.41) | | 0.178 | |
| Model 2 | | 0.00 (ref) | | –3.02 (–12.41, 6.38) | | –1.29 (–9.23, 6.64) | | | | **8.03 (0.57, 15.49)** | | | **0.009** | | |  | | **2.01 (0.12, 3.90)** | | **0.038** | |
| Model 3 | | 0.00 (ref) | | 0.11 (–10.31, 10.52) | | 0.33 (–8.63, 9.29) | | | | **12.42 (3.67, 21.16)** | | | **0.004** | | |  | | **2.71 (0.65, 4.78)** | | **0.015** | |
| BPF | |  | |  | |  | | | |  | | |  | | |  | |  | |  | |
| Model 1 | | 0.00 (ref) | | 8.33 (–1.02, 17.69) | | 4.58 (–3.50, 12.66) | | | | 5.95 (–4.59, 16.49) | | | 0.482 | | |  | | **1.44 (0.04, 2.85)** | | **0.045** | |
| Model 2 | | 0.00 (ref) | | 6.84 (–2.98, 16.67) | | 3.55 (–2.73, 9.83) | | | | 4.28 (–4.00, 12.55) | | | 0.576 | | |  | | 0.94 (–0.43, 2.31) | | 0.170 | |
| Model 3 | | 0.00 (ref) | | 2.15 (–9.51, 13.81) | | 0.80 (–6.83, 8.43) | | | | 0.10 (–9.70, 9.91) | | | 0.915 | | |  | | 0.65 (–0.87, 2.17) | | 0.365 | |
| ∑BPs | |  | |  | |  | | | |  | | |  | | |  | |  | |  | |
| Model 1 | | 0.00 (ref) | | 5.51 (–3.55, 14.57) | | 3.65 (–7.57, 14.87) | | | | 10.41 (–1.90, 22.72) | | | 0.140 | | |  | | **3.58 (1.37, 5.80)** | | **0.003** | |
| Model 2 | | 0.00 (ref) | | 4.90 (–3.24, 13.04) | | 3.71 (–5.00, 12.41) | | | | 10.15 (–0.35, 20.65) | | | 0.099 | | |  | | **2.98 (0.96, 5.00)** | | **0.006** | |
| Model 3 | | 0.00 (ref) | | 5.02 (–6.68, 16.71) | | 1.88 (–9.57, 13.33) | | | | 9.03 (–4.21, 22.27) | | | 0.184 | | |  | | **2.93 (0.41, 5.45)** | | **0.027** | |
| **Male** | |  | |  | |  | | | |  | | |  | | |  | |  | |  | |
| BPA | |  | |  | |  | | | |  | | |  | | |  | |  | |  | |
| Model 1 | | 0.00 (ref) | | 4.18 (–8.18, 16.54) | | 3.79 (–11.23, 18.81) | | | | 2.14 (–13.80, 18.07) | | | 0.998 | | |  | | 1.87 (–1.72, 5.46) | | 0.294 | |
| Model 2 | | 0.00 (ref) | | 2.27 (–9.79, 14.32) | | 0.11 (–14.95, 15.17) | | | | –2.62 (–16.73, 11.49) | | | 0.530 | | |  | | 0.79 (–2.49, 4.06) | | 0.625 | |
| Model 3 | | 0.00 (ref) | | –1.09 (–15.26, 13.08) | | 0.26 (–15.45, 15.98) | | | | –2.22 (–17.44, 13.01) | | | 0.740 | | |  | | 1.44 (–2.10, 4.97) | | 0.390 | |
| BPS | |  | |  | |  | | | |  | | |  | | |  | |  | |  | |
| Model 1 | | 0.00 (ref) | | –4.13 (–16.68, 8.42) | | 2.97 (–11.07, 17.01) | | | | 4.83 (–12.17, 21.83) | | | 0.428 | | |  | | 1.20 (–2.11, 4.51) | | 0.463 | |
| Model 2 | | 0.00 (ref) | | –8.48 (–20.22, 3.27) | | –1.71 (–13.96, 10.54) | | | | 3.35 (–11.85, 18.55) | | | 0.338 | | |  | | 1.16 (–1.96, 4.28) | | 0.451 | |
| Model 3 | | 0.00 (ref) | | –5.21 (–16.43, 6.00) | | –0.34 (–14.06, 13.38) | | | | 9.44 (–5.26, 24.15) | | | 0.098 | | |  | | 1.84 (–1.63, 5.32) | | 0.267 | |
| BPF | |  | |  | |  | | | |  | | |  | | |  | |  | |  | |
| Model 1 | | 0.00 (ref) | | 6.68 (–5.81, 19.17) | | 7.79 (–4.86, 20.44) | | | | 10.96 (–2.47, 24.39) | | | 0.182 | | |  | | 2.15 (–0.01, 4.31) | | 0.051 | |
| Model 2 | | 0.00 (ref) | | 4.40 (–9.20, 18.01) | | 9.07 (–1.86, 20.00) | | | | 8.80 (–4.64, 22.25) | | | 0.286 | | |  | | 1.60 (–0.61, 3.81) | | 0.150 | |
| Model 3 | | 0.00 (ref) | | 2.02 (–12.79, 16.83) | | 8.73 (–3.11, 20.58) | | | | 5.88 (–11.63, 23.38) | | | 0.599 | | |  | | 1.62 (–1.17, 4.41) | | 0.228 | |
| ∑BPs | |  | |  | |  | | | |  | | |  | | |  | |  | |  | |
| Model 1 | | 0.00 (ref) | | –5.48 (–17.36, 6.40) | | 4.30 (–11.07, 19.68) | | | | 4.01 (–11.28, 19.29) | | | 0.350 | | |  | | **3.51 (0.47, 6.55)** | | **0.025** | |
| Model 2 | | 0.00 (ref) | | –3.32 (–14.85, 8.22) | | 4.64 (–10.27, 19.56) | | | | 1.98 (–12.97, 16.93) | | | 0.672 | | |  | | 2.35 (–0.65, 5.35) | | 0.119 | |
| Model 3 | | 0.00 (ref) | | –0.45 (–14.29, 13.38) | | 8.27 (–7.78, 24.32) | | | | 4.92 (–13.54, 23.38) | | | 0.592 | | |  | | 3.53 (–0.85, 7.91) | | 0.103 | |
| **Female** | |  | |  | |  | | | |  | | |  | | |  | |  | |  | |
| BPA | |  | |  | |  | | | |  | | |  | | |  | |  | |  | |
| Model 1 | | 0.00 (ref) | | 0.48 (–14.71, 15.67) | | 12.42 (–2.80, 27.65) | | | | 2.29 (–13.81, 18.39) | | | 0.919 | | |  | | 2.67 (–0.33, 5.66) | | 0.079 | |
| Model 2 | | 0.00 (ref) | | –0.76 (–15.79, 14.28) | | 7.16 (–8.13, 22.46) | | | | –1.91 (–15.96, 12.15) | | | 0.659 | | |  | | 1.31 (–1.40, 4.03) | | 0.330 | |
| Model 3 | | 0.00 (ref) | | –4.5 (–20.98, 11.98) | | 3.56 (–17.79, 24.91) | | | | –10.67 (–29.57, 8.23) | | | 0.168 | | |  | | –0.62 (–4.25, 3.02) | | 0.717 | |
| BPS | |  | |  | |  | | | |  | | |  | | |  | |  | |  | |
| Model 1 | | 0.00 (ref) | | 4.23 (–7.11, 15.56) | | 3.35 (–7.76, 14.45) | | | | **15.78 (3.48, 28.07)** | | | **0.012** | | |  | | **3.82 (1.04, 6.60)** | | **0.009** | |
| Model 2 | | 0.00 (ref) | | 0.53 (–11.33, 12.38) | | –2.06 (–11.36, 7.24) | | | | 10.27 (–0.01, 20.55) | | | **0.020** | | |  | | **2.54 (0.02, 5.07)** | | **0.048** | |
| Model 3 | | 0.00 (ref) | | 4.27 (–8.79, 17.33) | | –0.11 (–11.19, 10.98) | | | | **13.62 (0.64, 26.60)** | | | **0.023** | | |  | | **3.39 (0.53, 6.25)** | | **0.024** | |
| BPF | |  | |  | |  | | | |  | | |  | | |  | |  | |  | |
| Model 1 | | 0.00 (ref) | | 9.85 (–2.49, 22.19) | | 5.16 (–8.84, 19.16) | | | | 1.12 (–10.86, 13.10) | | | 0.761 | | |  | | 0.82 (–1.47, 3.11) | | 0.471 | |
| Model 2 | | 0.00 (ref) | | 8.88 (–4.59, 22.34) | | 0.40 (–12.78, 13.58) | | | | –2.46 (–13.09, 8.17) | | | 0.353 | | |  | | 0.30 (–1.94, 2.55) | | 0.784 | |
| Model 3 | | 0.00 (ref) | | 3.15 (–11.65, 17.95) | | –5.43 (–22.67, 11.81) | | | | –8.38 (–19.70, 2.94) | | | 0.091 | | |  | | –0.49 (–2.77, 1.79) | | 0.647 | |
| ∑BPs | |  | |  | |  | | | |  | | |  | | |  | |  | |  | |
| Model 1 | | 0.00 (ref) | | 9.05 (–6.70, 24.80) | | 8.23 (–5.39, 21.85) | | | | **19.74 (4.66, 34.81)** | | | **0.025** | | |  | | **4.75 (1.69, 7.81)** | | **0.004** | |
| Model 2 | | 0.00 (ref) | | 3.74 (–10.12, 17.60) | | 1.56 (–10.60, 13.71) | | | | 13.31 (–1.19, 27.81) | | | 0.072 | | |  | | **3.45 (0.37, 6.54)** | | **0.030** | |
| Model 3 | | 0.00 (ref) | | 3.23 (–16.36, 22.83) | | –5.90 (–21.11, 9.32) | | | | 8.18 (–9.02, 25.39) | | | 0.252 | | |  | | 2.31 (–1.47, 6.09) | | 0.205 | |
| ^a^ The effect of bisphenols exposure on serum uric acid levels was expressed as the coefficient and its 95% confidence interval.  Abbreviations: BPA, bisphenol A; BPS, bisphenol S; BPF, bisphenol F; ∑BPs, the mass sum of the three bisphenols.  Model 1 was adjusted for urinary creatinine. Model 2 was adjusted for urinary creatinine, age, sex, BMI. Model 3 was adjusted for urinary creatinine, age, sex, BMI, ethnicity, smoking and drinking status, education, income, hyperlipidemia, diabetes, hypertension, and eGFR. | | | | | | | | | | | | | | | | | | | | | |
| Table S3. Associations of urinary bisphenols concentrations with hyperuricemia prevalence, excluding individuals with anti-gout treatment. ^a^ | | | | | | | | | | | | | | | | | | | |  |  |
| Outcomes | | Categorical models | | | | | | | | | | |  | | | Continuous models | | | |  |  |
|  |  | Quartile 1 | | Quartile 2 | | Quartile 3 | Quartile 4 | | | *P*_trend_ | | |  | | | Doubling change | | *P* value | |  |  |
| **Total** | |  | |  | |  |  | | |  | | |  | | |  | |  | |  |  |
| BPA | |  | |  | |  |  | | |  | | |  | | |  | |  | |  |  |
| Model 1 | | 1.00 (ref) | | **1.59 (1.14, 2.23)** | | **1.45 (1.08, 1.93)** | **1.42 (1.04, 1.93)** | | | 0.354 | | |  | | | **1.08 (1.01, 1.16)** | | **0.020** | |  |  |
| Model 2 | | 1.00 (ref) | | **1.71 (1.23, 2.38)** | | 1.37 (0.98, 1.93) | **1.38 (1.03, 1.84)** | | | 0.537 | | |  | | | 1.07 (1.00, 1.14) | | 0.064 | |  |  |
| Model 3 | | 1.00 (ref) | | **1.71 (1.07, 2.73)** | | 1.36 (0.88, 2.08) | 1.30 (0.91, 1.86) | | | 0.854 | | |  | | | 1.05 (0.97, 1.13) | | 0.225 | |  |  |
| BPS | |  | |  | |  |  | | |  | | |  | | |  | |  | |  |  |
| Model 1 | | 1.00 (ref) | | 1.09 (0.73, 1.62) | | 1.07 (0.76, 1.50) | 1.28 (0.89, 1.85) | | | 0.142 | | |  | | | 1.05 (0.97, 1.14) | | 0.210 | |  |  |
| Model 2 | | 1.00 (ref) | | 1.00 (0.64, 1.56) | | 0.94 (0.69, 1.29) | 1.26 (0.88, 1.81) | | | 0.094 | | |  | | | 1.05 (0.97, 1.13) | | 0.237 | |  |  |
| Model 3 | | 1.00 (ref) | | 1.04 (0.63, 1.71) | | 0.90 (0.62, 1.31) | 1.33 (0.83, 2.13) | | | 0.142 | | |  | | | 1.05 (0.96, 1.14) | | 0.258 | |  |  |
| BPF | |  | |  | |  |  | | |  | | |  | | |  | |  | |  |  |
| Model 1 | | 1.00 (ref) | | 1.21 (0.90, 1.63) | | 1.22 (0.88, 1.69) | 1.28 (0.90, 1.83) | | | 0.289 | | |  | | | **1.06 (1.00, 1.12)** | | **0.042** | |  |  |
| Model 2 | | 1.00 (ref) | | 1.18 (0.84, 1.65) | | 1.19 (0.83, 1.71) | 1.24 (0.86, 1.79) | | | 0.380 | | |  | | | 1.05 (0.99, 1.12) | | 0.132 | |  |  |
| Model 3 | | 1.00 (ref) | | 1.07 (0.66, 1.74) | | 1.15 (0.71, 1.85) | 1.10 (0.70, 1.73) | | | 0.764 | | |  | | | 1.04 (0.97, 1.12) | | 0.278 | |  |  |
| ∑BPs | |  | |  | |  |  | | |  | | |  | | |  | |  | |  |  |
| Model 1 | | 1.00 (ref) | | 1.20 (0.85, 1.70) | | 1.29 (0.91, 1.83) | 1.44 (0.93, 2.23) | | | 0.154 | | |  | | | **1.13 (1.04, 1.22)** | | **0.005** | |  |  |
| Model 2 | | 1.00 (ref) | | 1.15 (0.79, 1.66) | | 1.23 (0.86, 1.74) | 1.32 (0.83, 2.09) | | | 0.305 | | |  | | | **1.10 (1.01, 1.20)** | | **0.032** | |  |  |
| Model 3 | | 1.00 (ref) | | 1.12 (0.69, 1.84) | | 1.14 (0.74, 1.73) | 1.20 (0.69, 2.09) | | | 0.549 | | |  | | | 1.09 (0.99, 1.20) | | 0.085 | |  |  |
| **Male** | |  | |  | |  |  | | |  | | |  | | |  | |  | |  |  |
| BPA | |  | |  | |  |  | | |  | | |  | | |  | |  | |  |  |
| Model 1 | | 1.00 (ref) | | 1.62 (1.00, 2.61) | | 1.60 (0.96, 2.66) | 1.39 (0.86, 2.25) | | | 0.744 | | |  | | | 1.10 (1.00, 1.21) | | 0.051 | |  |  |
| Model 2 | | 1.00 (ref) | | 1.58 (0.98, 2.56) | | 1.45 (0.86, 2.45) | 1.22 (0.76, 1.96) | | | 0.819 | | |  | | | 1.07 (0.98, 1.18) | | 0.149 | |  |  |
| Model 3 | | 1.00 (ref) | | 1.51 (0.81, 2.79) | | 1.47 (0.81, 2.65) | 1.29 (0.73, 2.28) | | | 0.809 | | |  | | | 1.10 (0.98, 1.23) | | 0.096 | |  |  |
| BPS | |  | |  | |  |  | | |  | | |  | | |  | |  | |  |  |
| Model 1 | | 1.00 (ref) | | 0.98 (0.57, 1.68) | | 1.00 (0.60, 1.67) | 0.95 (0.56, 1.61) | | | 0.828 | | |  | | | 0.98 (0.88, 1.08) | | 0.633 | |  |  |
| Model 2 | | 1.00 (ref) | | 0.86 (0.48, 1.52) | | 0.87 (0.56, 1.36) | 0.91 (0.54, 1.51) | | | 0.857 | | |  | | | 0.98 (0.88, 1.08) | | 0.616 | |  |  |
| Model 3 | | 1.00 (ref) | | 0.92 (0.53, 1.60) | | 0.87 (0.55, 1.37) | 1.01 (0.60, 1.71) | | | 0.819 | | |  | | | 0.99 (0.89, 1.10) | | 0.784 | |  |  |
| BPF | |  | |  | |  |  | | |  | | |  | | |  | |  | |  |  |
| Model 1 | | 1.00 (ref) | | 0.96 (0.58, 1.60) | | 1.30 (0.75, 2.24) | 1.36 (0.83, 2.23) | | | 0.201 | | |  | | | **1.09 (1.00, 1.18)** | | **0.041** | |  |  |
| Model 2 | | 1.00 (ref) | | 0.89 (0.50, 1.57) | | 1.35 (0.79, 2.31) | 1.31 (0.78, 2.19) | | | 0.263 | | |  | | | 1.08 (0.99, 1.18) | | 0.097 | |  |  |
| Model 3 | | 1.00 (ref) | | 0.90 (0.43, 1.87) | | 1.45 (0.76, 2.77) | 1.28 (0.66, 2.49) | | | 0.423 | | |  | | | 1.09 (0.97, 1.22) | | 0.125 | |  |  |
| ∑BPs | |  | |  | |  |  | | |  | | |  | | |  | |  | |  |  |
| Model 1 | | 1.00 (ref) | | 0.74 (0.48, 1.14) | | 1.05 (0.65, 1.69) | 1.04 (0.60, 1.78) | | | 0.520 | | |  | | | 1.11 (0.99, 1.25) | | 0.078 | |  |  |
| Model 2 | | 1.00 (ref) | | 0.76 (0.47, 1.24) | | 1.06 (0.66, 1.72) | 0.98 (0.55, 1.75) | | | 0.779 | | |  | | | 1.08 (0.96, 1.22) | | 0.210 | |  |  |
| Model 3 | | 1.00 (ref) | | 0.75 (0.40, 1.41) | | 1.12 (0.66, 1.91) | 1.01 (0.51, 2.00) | | | 0.738 | | |  | | | 1.11 (0.96, 1.29) | | 0.149 | |  |  |
| **Female** | |  | |  | |  |  | | |  | | |  | | |  | |  | |  |  |
| BPA | |  | |  | |  |  | | |  | | |  | | |  | |  | |  |  |
| Model 1 | | 1.00 (ref) | | 1.12 (0.67, 1.88) | | **1.88 (1.17, 3.01)** | 1.08 (0.67, 1.77) | | | 0.847 | | |  | | | 1.09 (0.99, 1.19) | | 0.071 | |  |  |
| Model 2 | | 1.00 (ref) | | 1.15 (0.69, 1.93) | | **1.77 (1.06, 2.95)** | 0.96 (0.57, 1.61) | | | 0.491 | | |  | | | 1.06 (0.96, 1.16) | | 0.272 | |  |  |
| Model 3 | | 1.00 (ref) | | 1.06 (0.56, 1.99) | | 1.78 (0.89, 3.54) | 0.75 (0.37, 1.53) | | | 0.200 | | |  | | | 0.99 (0.88, 1.12) | | 0.876 | |  |  |
| BPS | |  | |  | |  |  | | |  | | |  | | |  | |  | |  |  |
| Model 1 | | 1.00 (ref) | | 1.29 (0.91, 1.83) | | 1.30 (0.88, 1.91) | 1.94 (1.19, 3.16) | | | **0.017** | | |  | | | **1.16 (1.05, 1.29)** | | **0.006** | |  |  |
| Model 2 | | 1.00 (ref) | | 1.18 (0.80, 1.74) | | 1.11 (0.75, 1.64) | 1.74 (1.06, 2.85) | | | **0.040** | | |  | | | **1.14 (1.03, 1.26)** | | **0.017** | |  |  |
| Model 3 | | 1.00 (ref) | | 1.16 (0.71, 1.90) | | 0.95 (0.56, 1.61) | 1.66 (0.84, 3.27) | | | 0.123 | | |  | | | 1.13 (0.99, 1.29) | | 0.070 | |  |  |
| BPF | |  | |  | |  |  | | |  | | |  | | |  | |  | |  |  |
| Model 1 | | 1.00 (ref) | | **1.54 (1.02, 2.31)** | | 1.21 (0.81, 1.80) | 1.12 (0.77, 1.64) | | | 0.960 | | |  | | | 1.02 (0.96, 1.09) | | 0.491 | |  |  |
| Model 2 | | 1.00 (ref) | | 1.59 (0.95, 2.67) | | 1.08 (0.68, 1.73) | 1.06 (0.71, 1.58) | | | 0.753 | | |  | | | 1.01 (0.94, 1.09) | | 0.753 | |  |  |
| Model 3 | | 1.00 (ref) | | 1.32 (0.64, 2.75) | | 0.94 (0.52, 1.70) | 0.78 (0.45, 1.35) | | | 0.222 | | |  | | | 0.97 (0.88, 1.07) | | 0.460 | |  |  |
| ∑BPs | |  | |  | |  |  | | |  | | |  | | |  | |  | |  |  |
| Model 1 | | 1.00 (ref) | | 1.60 (0.91, 2.82) | | 1.55 (0.95, 2.54) | **2.03 (1.23, 3.36)** | | | **0.035** | | |  | | | **1.16 (1.05, 1.28)** | | **0.006** | |  |  |
| Model 2 | | 1.00 (ref) | | 1.38 (0.80, 2.39) | | 1.24 (0.79, 1.95) | **1.77 (1.04, 2.99)** | | | 0.099 | | |  | | | **1.13 (1.01, 1.27)** | | **0.034** | |  |  |
| Model 3 | | 1.00 (ref) | | 1.32 (0.62, 2.82) | | 0.98 (0.57, 1.70) | 1.32 (0.67, 2.63) | | | 0.540 | | |  | | | 1.06 (0.92, 1.22) | | 0.374 | |  |  |
| ^a^ The effect of bisphenols exposure on hyperuricemia risk was expressed as the odds ratio and its 95% confidence interval.  Abbreviations: BPA, bisphenol A; BPS, bisphenol S; BPF, bisphenol F; ∑BPs, the mass sum of the three bisphenols.  Model 1 was adjusted for urinary creatinine. Model 2 was adjusted for urinary creatinine, age, sex, BMI. Model 3 was adjusted for urinary creatinine, age, sex, BMI, ethnicity, smoking and drinking status, education, income, hyperlipidemia, diabetes, hypertension, and eGFR. | | | | | | | | | | | | | | | | | | | |  |  |
| Table S4. Associations of urinary bisphenols concentrations with serum uric acid levels (μmol/L), adjusting drinks, organ and seafood intake. ^a^ | | | | | | | | | | | | | | | | | | | |  |  |
| Outcomes | Categorical models | | | | | | | | | | | | |  | | Continuous models | | | |  |  |
|  | Quartile 1 | | Quartile 2 | | Quartile 3 | | | Quartile 4 | | | *P*_trend_ | | |  | | Doubling change | | *P* value | |  |  |
| **Total** |  | |  | |  | | |  | | |  | | |  | |  | |  | |  |  |
| BPA | 0.00 (ref) | | –1.86 (–15.86, 12.14) | | –4.41 (–18.09, 9.27) | | | –6.49 (–21.30, 8.32) | | | 0.311 | | |  | | –0.47 (–3.39, 2.45) | | 0.719 | |  |  |
| BPS | 0.00 (ref) | | 1.68 (–10.43, 13.80) | | –0.05 (–9.83, 9.73) | | | **13.53 (2.99, 24.07)** | | | 0.496 | | |  | | **3.20 (0.86, 5.54)** | | **0.014** | |  |  |
| BPF | 0.00 (ref) | | 1.86 (–10.05, 13.76) | | 0.81 (–7.04, 8.65) | | | 1.43 (–10.73, 13.58) | | | 0.093 | | |  | | 0.72 (–1.30, 2.74) | | 0.433 | |  |  |
| ∑BPs | 0.00 (ref) | | 3.02 (–10.44, 16.47) | | 2.77 (–10.28, 15.83) | | | 8.27 (–7.55, 24.08) | | | **0.010** | | |  | | 2.87 (–0.54, 6.27) | | 0.088 | |  |  |
| **Male** |  | |  | |  | | |  | | |  | | |  | |  | |  | |  |  |
| BPA | 0.00 (ref) | | –7.16 (–23.30, 8.98) | | –0.88 (–20.93, 19.17) | | | –7.29 (–25.57, 10.99) | | | 0.112 | | |  | | 0.38 (–3.93, 4.70) | | 0.845 | |  |  |
| BPS | 0.00 (ref) | | –4.02 (–19.14, 11.11) | | –0.07 (–14.07, 13.94) | | | 10.51 (–7.14, 28.17) | | | **0.043** | | |  | | 2.37 (–1.50, 6.25) | | 0.199 | |  |  |
| BPF | 0.00 (ref) | | 1.79 (–16.30, 19.87) | | 9.73 (–3.27, 22.73) | | | 7.20 (–11.51, 25.91) | | | 0.856 | | |  | | 1.85 (–1.15, 4.85) | | 0.197 | |  |  |
| ∑BPs | 0.00 (ref) | | –1.07 (–15.80, 13.66) | | 10.34 (–8.74, 29.43) | | | 3.88 (–16.70, 24.45) | | | 0.517 | | |  | | 3.41 (–1.78, 8.60) | | 0.171 | |  |  |
| **Female** |  | |  | |  | | |  | | |  | | |  | |  | |  | |  |  |
| BPA | 0.00 (ref) | | –6.96 (–24.25, 10.32) | | –0.72 (–20.69, 19.25) | | | –15.24 (–35.31, 4.83) | | | 0.288 | | |  | | –1.42 (–4.89, 2.05) | | 0.379 | |  |  |
| BPS | 0.00 (ref) | | 6.10 (–7.66, 19.85) | | –1.74 (–16.41, 12.94) | | | 14.17 (–0.64, 28.97) | | | 0.228 | | |  | | **3.67 (0.28, 7.07)** | | **0.037** | |  |  |
| BPF | 0.00 (ref) | | 1.92 (–14.48, 18.32) | | –5.85 (–24.23, 12.53) | | | –6.55 (–22.12, 9.02) | | | 0.748 | | |  | | –0.43 (–3.30, 2.44) | | 0.744 | |  |  |
| ∑BPs | 0.00 (ref) | | –1.02 (–21.12, 19.09) | | –6.36 (–25.29, 12.58) | | | 6.43 (–14.05, 26.91) | | | 0.286 | | |  | | 2.27 (–2.31, 6.85) | | 0.291 | |  |  |
| ^a^ The effect of bisphenols exposure on serum uric acid levels was expressed as the coefficient and its 95% confidence interval.  Model was adjusted for urinary creatinine, age, sex, BMI, ethnicity, smoking and drinking status, education, income, hyperlipidemia, diabetes, hypertension, eGFR, drinks, organ and seafood intake. | | | | | | | | | | | | | | | | | | | |  |  |

| Table S5. Associations of urinary bisphenols concentrations with hyperuricemia prevalence, adjusting drinks, organ and seafood intake. ^a^ | | | | | | | | |
| --- | --- | --- | --- | --- | --- | --- | --- | --- |
| Outcomes | Categorical models | | | | |  | Continuous models | |
|  | Quartile 1 | Quartile 2 | Quartile 3 | Quartile 4 | *P*_trend_ |  | Doubling change | *P* value |
| **Total** |  |  |  |  |  |  |  |  |
| BPA | 1.00 (ref) | 1.48 (0.86, 2.56) | 1.25 (0.84, 1.87) | 1.09 (0.73, 1.63) | 0.594 |  | 1.01 (0.93, 1.11) | 0.724 |
| BPS | 1.00 (ref) | 1.14 (0.63, 2.07) | 0.86 (0.58, 1.29) | 1.40 (0.81, 2.42) | 0.161 |  | 1.05 (0.96, 1.16) | 0.244 |
| BPF | 1.00 (ref) | 1.02 (0.60, 1.73) | 1.13 (0.67, 1.90) | 1.03 (0.61, 1.71) | 0.993 |  | 1.02 (0.94, 1.11) | 0.555 |
| ∑BPs | 1.00 (ref) | 1.07 (0.67, 1.72) | 1.16 (0.75, 1.78) | 1.08 (0.63, 1.84) | 0.917 |  | 1.07 (0.95, 1.20) | 0.242 |
| **Male** |  |  |  |  |  |  |  |  |
| BPA | 1.00 (ref) | 1.16 (0.59, 2.28) | 1.42 (0.75, 2.70) | 1.07 (0.54, 2.11) | 0.921 |  | 1.07 (0.92, 1.25) | 0.330 |
| BPS | 1.00 (ref) | 1.03 (0.51, 2.09) | 0.90 (0.57, 1.43) | 1.05 (0.56, 1.98) | 0.851 |  | 0.99 (0.87, 1.11) | 0.800 |
| BPF | 1.00 (ref) | 0.86 (0.35, 2.12) | 1.45 (0.71, 2.97) | 1.20 (0.61, 2.38) | 0.580 |  | 1.08 (0.95, 1.22) | 0.217 |
| ∑BPs | 1.00 (ref) | 0.74 (0.36, 1.52) | 1.20 (0.66, 2.17) | 0.94 (0.43, 2.06) | 0.987 |  | 1.09 (0.92, 1.30) | 0.296 |
| **Female** |  |  |  |  |  |  |  |  |
| BPA | 1.00 (ref) | 1.07 (0.54, 2.13) | 1.63 (0.85, 3.14) | 0.62 (0.26, 1.45) | 0.103 |  | 0.95 (0.84, 1.09) | 0.440 |
| BPS | 1.00 (ref) | 1.23 (0.75, 2.04) | 0.80 (0.43, 1.49) | 1.67 (0.78, 3.56) | 0.147 |  | 1.13 (0.97, 1.32) | 0.099 |
| BPF | 1.00 (ref) | 1.24 (0.57, 2.70) | 0.98 (0.50, 1.91) | 0.73 (0.37, 1.43) | 0.180 |  | 0.96 (0.85, 1.08) | 0.442 |
| ∑BPs | 1.00 (ref) | 1.05 (0.52, 2.13) | 0.93 (0.51, 1.71) | 1.11 (0.54, 2.29) | 0.740 |  | 1.03 (0.88, 1.22) | 0.651 |
| ^a^ The effect of bisphenols exposure on hyperuricemia risk was expressed as the odds ratio and its 95% confidence interval.  Model was adjusted for urinary creatinine, age, sex, BMI, ethnicity, smoking and drinking status, education, income, hyperlipidemia, diabetes, hypertension, eGFR, drinks, organ and seafood intake. | | | | | | | | |

| Table S6. Associations of urinary bisphenols concentrations with gout prevalence, adjusting drinks, organ and seafood intake. ^a^ | | | | | | | | |
| --- | --- | --- | --- | --- | --- | --- | --- | --- |
| Outcomes | Categorical models | | | | |  | Continuous models | |
|  | Quartile 1 | Quartile 2 | Quartile 3 | Quartile 4 | *P*_trend_ |  | Doubling change | *P* value |
| **Total** |  |  |  |  |  |  |  |  |
| BPA | 1.00 (ref) | 0.70 (0.33, 1.48) | 0.72 (0.25, 2.06) | 0.66 (0.25, 1.71) | 0.453 |  | 0.87 (0.67, 1.12) | 0.226 |
| BPS | 1.00 (ref) | 1.45 (0.54, 3.86) | 0.84 (0.28, 2.56) | 2.23 (0.68, 7.27) | 0.172 |  | 1.14 (0.87, 1.48) | 0.300 |
| BPF | 1.00 (ref) | 0.59 (0.24, 1.43) | 0.84 (0.31, 2.31) | 0.39 (0.16, 0.98) | 0.054 |  | 0.94 (0.78, 1.13) | 0.428 |
| ∑BPs | 1.00 (ref) | 0.42 (0.17, 1.04) | 0.37 (0.11, 1.34) | 0.87 (0.23, 3.27) | 0.504 |  | 1.01 (0.73, 1.38) | 0.956 |
| **Male** |  |  |  |  |  |  |  |  |
| BPA | 1.00 (ref) | 0.32 (0.11, 0.88) | 0.50 (0.14, 1.77) | 0.98 (0.30, 3.28) | 0.448 |  | 0.87 (0.61, 1.24) | 0.383 |
| BPS | 1.00 (ref) | 1.56 (0.42, 5.76) | 0.95 (0.23, 3.87) | 1.25 (0.33, 4.80) | 0.912 |  | 0.99 (0.78, 1.26) | 0.921 |
| BPF | 1.00 (ref) | 0.63 (0.24, 1.66) | 0.79 (0.21, 3.03) | 0.43 (0.12, 1.50) | 0.196 |  | 0.93 (0.71, 1.21) | 0.547 |
| ∑BPs | 1.00 (ref) | 0.66 (0.20, 2.12) | 0.31 (0.11, 0.93) | 0.83 (0.19, 3.60) | 0.799 |  | 0.88 (0.58, 1.35) | 0.529 |
| **Female** |  |  |  |  |  |  |  |  |
| BPA | 1.00 (ref) | 1.69 (0.76, 3.79) | 0.62 (0.12, 3.28) | 0.39 (0.06, 2.45) | 0.155 |  | 0.80 (0.58, 1.10) | 0.144 |
| BPS | 1.00 (ref) | 0.97 (0.15, 6.50) | 0.99 (0.21, 4.63) | 6.00 (0.79, 45.84) | **0.045** |  | **1.52 (1.02, 2.25)** | **0.041** |
| BPF | 1.00 (ref) | 0.35 (0.05, 2.55) | 0.81 (0.24, 2.72) | 0.45 (0.08, 2.58) | 0.382 |  | 0.96 (0.70, 1.33) | 0.798 |
| ∑BPs | 1.00 (ref) | 0.50 (0.12, 2.15) | 0.82 (0.10, 6.71) | 1.71 (0.27, 10.89) | 0.211 |  | 1.23 (0.83, 1.82) | 0.256 |
| ^a^ The effect of bisphenols exposure on gout risk was expressed as the odds ratio and its 95% confidence interval.  Model was adjusted for urinary creatinine, age, sex, BMI, ethnicity, smoking and drinking status, education, income, hyperlipidemia, diabetes, hypertension, eGFR, drinks, organ and seafood intake. | | | | | | | | |

| Table S7. Associations of urinary bisphenols concentrations with eGFR levels. ^a^ | | | | | | | | |
| --- | --- | --- | --- | --- | --- | --- | --- | --- |
| Outcomes | Categorical models | | | | |  | Continuous models | |
|  | Quartile 1 | Quartile 2 | Quartile 3 | Quartile 4 | *P*_trend_ |  | Doubling change | *P* value |
| **Total** |  |  |  |  |  |  |  |  |
| BPA | 0.00 (ref) | –3.33 (–6.04, –0.61) | –1.23 (–3.78, 1.32) | –1.57 (–3.19, 0.06) | 0.700 |  | –0.13 (–0.53, 0.28) | 0.506 |
| BPS | 0.00 (ref) | **1.60 (0.98, 4.19)** | **2.43 (0.87, 5.73)** | **3.36 (0.70, 6.01)** | **0.023** |  | 0.54 (–0.05, 1.12) | 0.070 |
| BPF | 0.00 (ref) | –3.18 (–5.34, –1.03) | –1.85 (3.74, 0.04) | –2.68 (–4.98, –0.38) | 0.084 |  | –0.35 (–0.74, 0.04) | 0.075 |
| ∑BPs | 0.00 (ref) | –1.12 (–4.21, 1.96) | –0.80 (–3.60, 1.99) | –0.01 (–2.85, 2.83) | 0.545 |  | –0.14 (–0.73, 0.45) | 0.610 |
| **Male** |  |  |  |  |  |  |  |  |
| BPA | 0.00 (ref) | –3.53 (–6.35, –0.71) | –0.81 (3.24, 1.62) | –1.03 (–3.45, 1.39) | 0.607 |  | 0.09 (–0.49, 0.66) | 0.749 |
| BPS | 0.00 (ref) | **1.38 (1.39, 4.16)** | **0.26 (3.12, 3.63)** | **3.79 (0.73, 6.84)** | **0.026** |  | 0.43 (–0.15, 1.01) | 0.131 |
| BPF | 0.00 (ref) | –3.66 (–6.75, –0.58) | –2.45 (–5.49, 0.60) | –2.73 (–5.99, 0.54) | 0.278 |  | –0.35 (–0.93, 0.24) | 0.219 |
| ∑BPs | 0.00 (ref) | **0.53 (2.77, 3.84)** | **0.89 (1.54, 3.31)** | **1.55 (1.70, 4.80)** | 0.353 |  | 0.13 (–0.65, 0.91) | 0.731 |
| **Female** |  |  |  |  |  |  |  |  |
| BPA | 0.00 (ref) | –1.50 (5.02, 2.02) | –1.64 (–5.4, 2.12) | –1.84 (–5.18, 1.50) | 0.381 |  | –0.38 (–1.11, 0.34) | 0.271 |
| BPS | 0.00 (ref) | 1.47 (–2.65, 5.58) | 4.20 (0.60, 7.79) | 3.28 (–0.76, 7.31) | 0.133 |  | 0.67 (–0.14, 1.47) | 0.095 |
| BPF | 0.00 (ref) | –3.03 (–5.86, –0.20) | –1.24 (–4.39, 1.90) | –2.72 (–5.83, 0.39) | 0.184 |  | –0.39 (–0.96, 0.19) | 0.167 |
| ∑BPs | 0.00 (ref) | –1.91 (–5.30, 1.48) | –2.48 (–6.73, 1.78) | –2.04 (–6.51, 2.44) | 0.591 |  | –0.43 (–1.28, 0.41) | 0.284 |
| ^a^ The effect of bisphenols exposure on eGFR levels was expressed as the coefficient and its 95% confidence interval.  Model was adjusted for urinary creatinine, age, sex, BMI, ethnicity, smoking and drinking status, education, income, hyperlipidemia, diabetes, and hypertension. | | | | | | | | |

| Table S8. Mediating effects of eGFR on the associations between urinary bisphenols and serum uric acid levels (μmol/L). ^a^ | | | |
| --- | --- | --- | --- |
| Outcomes | Total effect | Direct effect | Mediating effect (*P* value) |
| **Total** |  |  |  |
| BPA | –0.12 (–224, 245.39) | 0.07 (–219, 234.44) | 0.01 (0.99) |
| BPS | 1.97 (–262, 237.2) | 2.86 (–255, 245.44) | 0.01 (0.90) |
| BPF | 1.33 (–151, 153.7) | 1.17 (–149, 151) | 0.01 (0.97) |
| ∑BPs | –2.4 (–282, 277.02) | –2.44 (–284, 282.49) | 0.01 (0.97) |
| **Male** |  |  |  |
| BPA | 23.5 (–646.75, 740.41) | 28.94 (–632.77, 714.78) | 0.02 (0.88) |
| BPS | –1.53 (–1000, 927.97) | –2.66 (–1010, 906.75) | 0.01 (0.90) |
| BPF | –2.02 (–733.32, 683.45) | –2.66 (–609.57, 616.41) | 0.07 (0.73) |
| ∑BPs | –11.7 (–1200, 1118) | –11.2 (–1110, 1035.58) | 0.05 (0.77) |
| **Female** |  |  |  |
| BPA | 6.49 (–789.99, 818.45) | 5.51 (–721.16, 708.39) | 0.04 (0.87) |
| BPS | 1.75 (–915.7, 899.3) | –0.69 (–848.26, 863.5) | 0.04 (0.85) |
| BPF | 5.78 (–506.34, 499.39) | 4.28 (–435.76, 424.59) | 0.09 (0.75) |
| ∑BPs | 18.3 (–1140, 1242.2) | 13.9 (–1070, 1162.4) | 0.04 (0.84) |
| ^a^ The effect of bisphenols exposure on serum uric acid levels was expressed as the coefficient and its 95% confidence interval.  Model was adjusted for urinary creatinine, age, sex, BMI, ethnicity, smoking and drinking status, education, income, hyperlipidemia, diabetes, and hypertension. | | | |

| Table S9. Sex difference of the study population, NHANES 2013-2016. | | | |
| --- | --- | --- | --- |
| Characteristics | Female  (N =1841) | Male  (N =1646) | *P* value |
| Age, mean (SE), year | 48.2±0.6 | 46.9±0.6 | **0.043** |
| Race/ethnicity |  |  | 0.082 |
| Non-Hispanic black | 417 (12.3) | 357 (10.3) |  |
| Non-Hispanic white | 657 (63.6) | 618 (65.1) |  |
| Mexican American | 275 (8.4) | 246 (9.4) |  |
| Other | 492 (15.7) | 425 (15.2) |  |
| Educational level |  |  | **0.019** |
| Less than high school | 173 (4.9) | 148 (5.2) |  |
| High school or GED | 619 (29.2) | 637 (34.0) |  |
| College or above | 1046 (65.9) | 860 (60.8) |  |
| BMI, kg/m^2^ |  |  | **<0.001** |
| <25 | 604 (37.3) | 470 (28.4) |  |
| 25-30 | 493 (28.2) | 630 (38.0) |  |
| ≥30 | 670 (34.5) | 507 (33.6) |  |
| IPR (family income to poverty ratio) | |  | 0.094 |
| <1.30 | 588 (24.3) | 469 (20.9) |  |
| 1.30-3.49 | 600 (35.0) | 545 (34.5) |  |
| ≥3.50 | 484 (40.8) | 480 (44.6) |  |
| Smoking status |  |  | **< 0.001** |
| Never | 1224 (63.2) | 791 (49.8) |  |
| Former | 301 (18.5) | 480 (28.8) |  |
| Current | 314 (18.3) | 372 (21.4) |  |
| Drinking status |  |  | **< 0.001** |
| Never | 361 (15.4) | 149 (7.5) |  |
| Former | 274 (14.2) | 272 (14.5) |  |
| Mild | 487 (30.7) | 592 (42.6) |  |
| Moderate | 291 (22.3) | 165 (12.9) |  |
| Heavy | 249 (17.3) | 343 (22.5) |  |
| Physical Activity, MET-minutes/week | |  | **0.001** |
| <600 | 300 (22.6) | 196 (14.8) |  |
| ≥600 | 974 (77.4) | 1092 (85.2) |  |
| Hyperlipidemia |  |  | 0.400 |
| No | 559 (31.9) | 558 (33.9) |  |
| Yes | 1282 (68.1) | 1088 (66.1) |  |
| Diabetes |  |  | 0.317 |
| No | 1453 (85.6) | 1302 (84.3) |  |
| Yes | 345 (14.4) | 344 (15.7) |  |
| Hypertension |  |  | 0.126 |
| No | 1055 (61.6) | 916 (58.6) |  |
| Yes | 786 (38.4) | 730 (41.4) |  |
| Hyperuricemia |  |  | **0.005** |
| No | 1465 (83.0) | 1202 (76.7) |  |
| Yes | 320 (17.0) | 372 (23.3) |  |
| Gout |  |  |  |
| No | 1792 (97.6) | 1540 (93.6) | **< 0.001** |
| Yes | 49 (2.4) | 106 (6.4) |  |
| eGFR, mL/min/1.73 m^2^ |  |  | **0.023** |
| <60 | 146 (8.0) | 118 (4.9) |  |
| 60-90 | 525 (32.2) | 541 (36.0) |  |
| ≥90 | 1114 (59.8) | 914 (59.1) |  |
| BPA, μg/L | 1.0 (0.5, 2.0) | 1.3 (0.6, 2.6) | **< 0.001** |
| BPS, μg/L | 0.4 (0.2, 1.0) | 0.5 (0.2, 1.1) | **0.006** |
| BPF, μg/L | 0.2 (0.1, 0.8) | 0.3 (0.1, 1.1) | **0.012** |
| ∑BPs, μg/L | 2.4 (1.2, 4.8) | 3.0 (1.6, 6.3) | **< 0.001** |
| Serum uric acid, μmol/L | 2.8 (2.4, 3.3) | 3.5 (3.1, 4.1) | **< 0.001** |
